# Supplementary material for: Application of MALDI-TOF MS and machine learning for the detection of SARS-CoV-2 and non-SARS-CoV-2 respiratory infections
Source: Microbiol Spectr. 2024 Mar 18;12(5):e04068-23. doi: 10.1128/spectrum.04068-23 (PMC11064577; doi:10.1128/spectrum.04068-23)
Supplement: Supplemental material — Figures S1 to S10; Tables S1 to S6. [file spectrum.04068-23-s0001.pdf]

Application of MALDI-TOF-MS and Machine Learning for the Detection of SARS-CoV-2 and non-SARS-CoV-2 Respiratory Infections.

Sergey Yegorov[1,2*], Irina Kadyrova[3*], Ilya Korshukov[3], Aidana Sultanbekova[3*], Yevgeniya Kolesnikova [3], Valentina Barkhanskaya [3], Tatiana Bashirova [6], Yerzhan Zhunusov[7], Yevgeniya Li [7], Viktoriya Parakhina[7,8], Svetlana Kolesnichenko[3], Yeldar Baiken[2,4,5], Bakhyt Matkarimov[4], Dmitriy Vazenmiller[3], Matthew S. Miller[1], Gonzalo H. Hortelano[2], Anar Turmukhambetova[3], Antonella E. Chesca [9], Dmitriy Babenko[3].

SUPPLEMENTARY

Table S1. Mass spectrometry peaks selected for ML training using the Nachtigall et al. strategy (see Methods) in the South American (SA) samples (Analysis I). Shown are peak median intensities and interquartile ranges (IQR). A total of 88 peaks identical to those used by Nachtigall et al were detected and then used in Analysis I (See Table S2 for the comparison of these same 88 peaks across the dataset from Kazakhstan). p-values were calculated using the two-tailed Wilcoxon rank sum test. NCARI= non-COVID acute respiratory infection.

South American samples			
**Peak (*m/z*)**	**NCARI, N = 151**	**SARS-CoV-2+, N = 211**	**p-value**
3044	1.1e-04 [6.1e-05; 1.8e-04]	6.5e-05 [1.9e-05; 1.3e-04]	<0.001***
3095	2.3e-04 [1.3e-04; 4.3e-04]	1.2e-04 [6.2e-05; 2.2e-04]	<0.001***
3112	1.6e-04 [8.4e-05; 2.5e-04]	1.1e-04 [5.0e-05; 1.8e-04]	<0.001***
3139	1.5e-04 [7.4e-05; 2.5e-04]	1.6e-04 [5.6e-05; 2.6e-04]	0.8
3152	1.2e-04 [3.4e-05; 2.5e-04]	2.3e-04 [1.2e-04; 4.6e-04]	<0.001***
3193	5.5e-05 [6.9e-06; 1.6e-04]	4.3e-05 [1.1e-05; 1.1e-04]	0.2
3242	1.0e-04 [5.9e-05; 1.5e-04]	1.1e-04 [6.5e-05; 1.7e-04]	0.3
3256	8.6e-05 [3.4e-05; 1.4e-04]	1.1e-04 [3.9e-05; 2.2e-04]	0.023*
3297	1.2e-04 [3.4e-05; 2.7e-04]	1.4e-04 [4.1e-05; 3.0e-04]	0.6
3318	9.6e-05 [1.3e-05; 3.2e-04]	1.7e-04 [1.2e-05; 3.4e-04]	0.5
3337	3.0e-04 [1.2e-04; 5.4e-04]	1.7e-04 [2.6e-05; 3.2e-04]	<0.001***
3358	2.3e-04 [1.3e-04; 3.4e-04]	9.9e-05 [4.1e-05; 1.7e-04]	<0.001***
3372	2e-03 [6.6e-04; 3.2e-03]	1e-03 [4.8e-04; 2.5e-03]	0.002**
3392	3.4e-04 [1.8e-04; 5.6e-04]	3.9e-04 [1.7e-04; 7.1e-04]	0.2
3443	2.4e-03 [7.4e-04; 3.9e-03]	1.3e-03 [5.3e-04; 3.2e-03]	0.010*
3464	6.6e-04 [3.2e-04; 1.3e-03]	4.6e-04 [2.4e-04; 8.5e-04]	0.010**
3476	3.8e-04 [2.4e-04; 6.2e-04]	3.5e-04 [2.0e-04; 5.5e-04]	0.032*
3487	1.2e-03 [5.9e-04; 2.8e-03]	5.9e-04 [2.7e-04; 1.4e-03]	<0.001***
3516	4.1e-05 [0e+00; 2.6e-04]	5.6e-05 [0e+00; 1.7e-04]	0.9
3609	1.1e-04 [4.1e-05; 1.8e-04]	1.1e-04 [4.2e-05; 1.7e-04]	0.4
3651	1.1e-04 [5.5e-05; 1.8e-04]	8.5e-05 [3.7e-05; 1.5e-04]	0.022*
3710	3.6e-04 [1.9e-04; 6.9e-04]	2.7e-04 [1.1e-04; 6.8e-04]	0.061
3732	9.7e-05 [2.3e-05; 1.8e-04]	8.7e-05 [3.8e-05; 1.5e-04]	0.8
3754	7.6e-05 [8.5e-06; 1.6e-04]	1.1e-04 [1.7e-05; 2.3e-04]	0.020*
3779	1.5e-04 [3.5e-05; 3.1e-04]	1.5e-04 [6.6e-05; 2.8e-04]	0.8
3792	1.4e-04 [5.9e-05; 3.7e-04]	1.4e-04 [4.2e-05; 3.0e-04]	0.4
3804	1.5e-04 [3.4e-05; 3.2e-04]	1.6e-04 [4.0e-05; 2.9e-04]	0.7
3827	1.6e-04 [5.2e-05; 3.8e-04]	1.4e-04 [4.5e-05; 2.7e-04]	0.12
3915	1.4e-04 [8.0e-05; 1.9e-04]	1.1e-04 [6.1e-05; 1.8e-04]	0.015*
3934	1.4e-04 [4.7e-05; 2.5e-04]	1.5e-04 [6.8e-05; 2.3e-04]	0.5
3981	1.2e-04 [5.6e-05; 2.2e-04]	1.7e-04 [1.1e-04; 2.6e-04]	<0.001***
4138	3.2e-04 [1.7e-04; 7.2e-04]	2.0e-04 [1.0e-04; 3.9e-04]	<0.001***
4160	2.5e-04 [1.0e-04; 7.0e-04]	1.6e-04 [8.6e-05; 3.2e-04]	<0.001***
4192	1.8e-04 [4.5e-05; 4.3e-04]	2.2e-04 [3.9e-05; 7.1e-04]	0.2
4229	1.4e-04 [5.6e-05; 3.2e-04]	1.5e-04 [8.7e-05; 2.7e-04]	0.5
4356	2.8e-04 [1.7e-04; 4.1e-04]	2.9e-04 [1.9e-04; 4.1e-04]	0.5
4374	2.1e-04 [1.2e-04; 3.9e-04]	2.1e-04 [1.2e-04; 6.2e-04]	0.6
4393	1.5e-04 [8.4e-05; 2.7e-04]	1.2e-04 [5.2e-05; 2.7e-04]	0.2
4428	7.5e-05 [2.0e-05; 1.7e-04]	7.0e-05 [2.1e-05; 1.3e-04]	0.5
4473	9.9e-05 [4.4e-05; 1.5e-04]	9.0e-05 [3.9e-05; 1.4e-04]	0.3
4532	5.7e-05 [1.8e-05; 1.3e-04]	1.3e-04 [8.4e-05; 2.1e-04]	<0.001***
4551	1.8e-04 [8.3e-05; 5.6e-04]	2.4e-04 [9.1e-05; 3.7e-04]	0.8
4574	1.1e-04 [4.1e-05; 1.7e-04]	1.4e-04 [4.6e-05; 2.3e-04]	0.032*
4636	9.3e-05 [4.9e-05; 1.8e-04]	1.2e-04 [7.0e-05; 2.0e-04]	0.021*
4715	9.6e-05 [4.5e-05; 2.0e-04]	1.2e-04 [6.1e-05; 2.2e-04]	0.11
4738	5.7e-05 [1.7e-05; 1.6e-04]	6.0e-05 [2.0e-05; 1.4e-04]	0.8
4811	5.3e-05 [1.4e-05; 1.2e-04]	5.0e-05 [2.3e-05; 8.8e-05]	0.4
4842	1.3e-04 [5.2e-05; 2.4e-04]	1.2e-04 [5.7e-05; 2.4e-04]	0.9
4901	9.0e-05 [2.5e-05; 2.1e-04]	1.3e-04 [5.5e-05; 2.5e-04]	0.012*
4940	2.5e-04 [1.2e-04; 4.6e-04]	2.1e-04 [1.3e-04; 3.8e-04]	0.6
4966	1.0e-03 [5.6e-04; 2.2e-03]	6.4e-04 [3.8e-04; 1.4e-03]	<0.001***
4986	8.0e-04 [4.2e-04; 1.4e-03]	6.9e-04 [3.4e-04; 1.3e-03]	0.3
5005	4.2e-04 [2.1e-04; 7.5e-04]	3.6e-04 [1.6e-04; 7.0e-04]	0.2
5047	9.1e-05 [2.3e-05; 2.0e-04]	7.7e-05 [1.6e-05; 1.6e-04]	0.3
5147	5.7e-05 [8.6e-06; 2.2e-04]	6.0e-05 [9.2e-06; 1.8e-04]	0.5
5218	1.7e-04 [7.9e-05; 3.0e-04]	1.3e-04 [5.1e-05; 2.8e-04]	0.036*
5236	2.9e-04 [8.0e-05; 7.6e-04]	1.4e-04 [4.9e-05; 5.8e-04]	0.005**
5256	1.1e-04 [2.9e-05; 2.6e-04]	8.1e-05 [2.7e-05; 2.1e-04]	0.2
5285	4.1e-05 [2.4e-06; 1.5e-04]	4.7e-05 [1.6e-05; 1.1e-04]	0.3

5382	2.1e-04 [1.0e-04; 3.7e-04]	1.6e-04 [7.2e-05; 3.0e-04]	0.012*
5402	1.4e-04 [7.2e-05; 2.5e-04]	1.3e-04 [7.2e-05; 2.8e-04]	0.9
5423	2.6e-04 [1.3e-04; 5.3e-04]	2.1e-04 [1.2e-04; 4.3e-04]	0.11
5530	7.0e-05 [1.6e-05; 1.8e-04]	5.7e-05 [1.5e-05; 1.7e-04]	0.6
5594	1.1e-04 [3.1e-05; 1.8e-04]	9.6e-05 [4.6e-05; 3.4e-04]	0.10
5869	2.2e-04 [1.4e-04; 5.3e-04]	2.5e-04 [1.3e-04; 4.9e-04]	0.9
5950	5.2e-05 [2.1e-05; 1.0e-04]	7.8e-05 [3.1e-05; 1.5e-04]	0.004**
6192	1.6e-04 [1.2e-04; 2.3e-04]	2.3e-04 [1.1e-04; 3.7e-04]	<0.001***
6361	1.2e-04 [4.5e-05; 2.3e-04]	8.9e-05 [3.4e-05; 1.6e-04]	0.008**
6639	8.8e-05 [4.2e-05; 1.6e-04]	9.0e-05 [5.0e-05; 1.5e-04]	0.9
6964	1.1e-04 [3.6e-05; 2.9e-04]	7.1e-05 [2.2e-05; 2.0e-04]	0.002**
7349	5.2e-04 [2.0e-04; 8.9e-04]	5.3e-04 [2.6e-04; 1.1e-03]	0.3
7612	1.6e-04 [5.5e-05; 4.6e-04]	7.7e-05 [2.3e-05; 1.9e-04]	<0.001***
7654	5.2e-05 [1.9e-05; 1.1e-04]	3.4e-05 [1.2e-05; 7.3e-05]	0.005**
7765	2.6e-05 [6.3e-06; 6.1e-05]	3.5e-05 [8.1e-06; 7.4e-05]	0.2
8215	3.5e-05 [1.3e-05; 8.2e-05]	6.1e-05 [3.1e-05; 1.1e-04]	<0.001***
8452	9.7e-05 [5.1e-05; 1.7e-04]	1.3e-04 [7.3e-05; 2.4e-04]	0.007**
8469	5.2e-05 [2.5e-05; 8.8e-05]	6.2e-05 [2.5e-05; 1.1e-04]	0.2
8568	1.6e-04 [9.9e-05; 3.2e-04]	1.6e-04 [7.6e-05; 2.9e-04]	0.5
8589	5.5e-05 [2.6e-05; 1.0e-04]	4.8e-05 [8.8e-06; 1.1e-04]	0.4
8742	3.3e-05 [1.2e-05; 6.7e-05]	4.0e-05 [1.5e-05; 7.6e-05]	0.11
9956	5.8e-05 [3.4e-05; 1.0e-04]	8.2e-05 [4.9e-05; 1.2e-04]	<0.001***
10096	7.7e-05 [3.9e-05; 1.5e-04]	8.9e-05 [4.2e-05; 1.6e-04]	0.5
10116	4.3e-05 [1.9e-05; 9.9e-05]	6.9e-05 [3.5e-05; 1.3e-04]	0.002**
10444	1.6e-04 [7.6e-05; 2.8e-04]	1.0e-04 [5.1e-05; 1.5e-04]	<0.001***
10837	2.5e-04 [1.1e-04; 6.4e-04]	1.8e-04 [1.1e-04; 4.5e-04]	0.085
11011	2.6e-05 [2.7e-06; 1.1e-04]	9.7e-05 [3.3e-05; 1.5e-04]	<0.001***
11735	1.8e-04 [6.0e-05; 2.6e-04]	1.6e-04 [7.9e-05; 3.2e-04]	0.5
14692	2.7e-04 [1.9e-04; 5.1e-04]	3.5e-04 [1.7e-04; 6.5e-04]	0.024*

Table S2. Mass spectrometry peaks identified using the Nachtigall et al. strategy (see Methods) in the Kazakhstan samples (Analysis I). Shown are peak median intensities and interquartile ranges (IQR). A total of 88 peaks identical to those used by Nachtigall et al were detected and then used in Analysis I (See Table S1 for the comparison of these same 88 peaks for the South American samples). p-values were calculated using the two-tailed Wilcoxon rank sum test. NCARI= non-COVID acute respiratory infection. AC=asymptomatic controls.

Kazakhstan samples					
**Peak (*m/z*)**	**NCARI, N = 98**	**AC, N = 39**	**SARS-CoV-2/2020, N = 108**	**SARS-CoV-2/2022, N = 7**	**p-value**
3044	4.5e-05 [2.3e-05; 8.1e-05]	7.2e-05 [2.7e-05; 1.1e-04]	8.4e-05 [4.8e-05; 1.1e-04]	1.5e-04 [7.7e-05; 1.9e-04]	<0.001***
3095	8.4e-05 [4.5e-05; 1.3e-04]	1.1e-04 [7.6e-05; 1.5e-04]	1.1e-04 [7.5e-05; 1.4e-04]	1.1e-04 [5.7e-05; 2.0e-04]	0.035*
3112	5.9e-05 [2.4e-05; 1.2e-04]	9.6e-05 [8.2e-05; 1.3e-04]	9.0e-05 [5.2e-05; 1.3e-04]	1.2e-05 [3.1e-06; 3.1e-05]	<0.001***
3139	1.6e-04 [1.2e-04; 2.4e-04]	1.1e-04 [7.7e-05; 1.4e-04]	1.1e-04 [8.2e-05; 1.7e-04]	1.8e-04 [1.2e-04; 2.3e-04]	<0.001***
3152	5.4e-05 [1.6e-05; 1.5e-04]	8.5e-05 [3.2e-05; 1.3e-04]	7.5e-05 [4.4e-05; 1.1e-04]	2.1e-05 [1.9e-06; 1.5e-04]	0.7
3193	1.2e-04 [7.2e-05; 1.9e-04]	1.1e-04 [8.1e-05; 1.3e-04]	1.2e-04 [9.5e-05; 1.9e-04]	1.5e-04 [1.2e-04; 1.6e-04]	0.046*
3242	8.8e-05 [5.3e-05; 1.4e-04]	1.1e-04 [7.9e-05; 1.4e-04]	1.1e-04 [8.9e-05; 1.6e-04]	7.5e-05 [5.6e-05; 1.7e-04]	0.008**
3256	9.7e-05 [5.2e-05; 1.5e-04]	1.1e-04 [7.5e-05; 1.5e-04]	7.1e-05 [4.0e-05; 1.2e-04]	1.2e-04 [7.6e-05; 1.3e-04]	0.005**
3297	1.6e-04 [1.0e-04; 2.8e-04]	1.1e-04 [6.9e-05; 1.3e-04]	1.0e-04 [7.2e-05; 1.4e-04]	1.9e-04 [7.3e-05; 2.8e-04]	<0.001***
3318	1.1e-04 [4.4e-05; 1.6e-04]	1.0e-04 [3.9e-05; 1.6e-04]	6.8e-05 [1.9e-05; 1.0e-04]	1.4e-04 [1.1e-04; 2.2e-04]	<0.001***
3337	1.6e-04 [7.5e-05; 4.5e-04]	3.3e-04 [1.6e-04; 4.3e-04]	1.5e-04 [9.9e-05; 2.7e-04]	2.3e-04 [1.8e-04; 4.0e-04]	0.019*
3358	1.4e-04 [7.6e-05; 2.0e-04]	1.1e-04 [7.3e-05; 1.6e-04]	8.9e-05 [5.3e-05; 1.3e-04]	1.3e-04 [8.0e-05; 1.7e-04]	<0.001***
3372	6.5e-04 [2.4e-04; 3.0e-03]	4.8e-04 [2.5e-04; 1.8e-03]	3.8e-04 [1.8e-04; 1.9e-03]	1.0e-03 [2.0e-04; 1.8e-03]	0.3
3392	1.6e-04 [8.1e-05; 3.1e-04]	9.5e-05 [6.6e-05; 1.3e-04]	1.1e-04 [5.8e-05; 1.8e-04]	1.9e-04 [1.1e-04; 2.8e-04]	0.004**
3443	7.2e-04 [2.2e-04; 3.3e-03]	9.7e-04 [2.5e-04; 2.8e-03]	5.7e-04 [2.7e-04; 2.9e-03]	1.3e-03 [2.3e-04; 1.6e-03]	0.9
3464	3.9e-04 [2.0e-04; 8.3e-04]	1.8e-04 [1.5e-04; 3.0e-04]	1.9e-04 [1.3e-04; 5.6e-04]	4.9e-04 [1.3e-04; 9.0e-04]	<0.001***
3476	5.2e-04 [1.8e-04; 8.7e-04]	3.5e-04 [2.3e-04; 5.1e-04]	3.4e-04 [1.8e-04; 5.4e-04]	2.7e-04 [1.5e-04; 9.3e-04]	0.048*
3487	7.3e-04 [3.0e-04; 2.0e-03]	5.7e-04 [1.8e-04; 1.8e-03]	3.8e-04 [1.6e-04; 1.1e-03]	7.8e-04 [2.0e-04; 1.6e-03]	0.027*
3516	8.7e-05 [4.1e-05; 1.5e-04]	1.1e-04 [7.6e-05; 1.4e-04]	1.1e-04 [7.7e-05; 1.6e-04]	2.4e-04 [1.6e-04; 3.1e-04]	0.034*
3609	1.5e-04 [1.1e-04; 2.1e-04]	7.6e-05 [4.7e-05; 1.1e-04]	1.0e-04 [5.4e-05; 1.3e-04]	2.2e-04 [1.7e-04; 3.4e-04]	<0.001***
3651	8.6e-05 [2.9e-05; 1.3e-04]	1.1e-04 [8.1e-05; 1.3e-04]	1.2e-04 [8.8e-05; 1.5e-04]	1.1e-04 [7.9e-05; 1.3e-04]	0.004**
3710	3.5e-04 [1.5e-04; 5.1e-04]	1.4e-04 [9.7e-05; 2.3e-04]	1.4e-04 [8.2e-05; 2.3e-04]	4.6e-04 [1.8e-04; 6.3e-04]	<0.001***
3732	1.6e-04 [1.1e-04; 3.0e-04]	1.5e-04 [1.1e-04; 2.0e-04]	9.1e-05 [6.0e-05; 1.3e-04]	8.8e-05 [8.0e-05; 2.2e-04]	<0.001***
3754	1.2e-04 [6.1e-05; 1.8e-04]	8.1e-05 [5.4e-05; 1.1e-04]	1.5e-04 [1.1e-04; 2.0e-04]	2.1e-04 [1.2e-04; 2.4e-04]	<0.001***
3779	6.6e-05 [2.0e-05; 1.2e-04]	1.0e-04 [5.2e-05; 1.4e-04]	1.1e-04 [7.1e-05; 1.7e-04]	3.0e-05 [1.2e-06; 4.9e-05]	<0.001***
3792	4.7e-05 [1.2e-05; 1.1e-04]	7.3e-05 [4.8e-05; 1.0e-04]	6.5e-05 [2.9e-05; 1.0e-04]	1.4e-05 [4.8e-06; 8.3e-05]	0.3
3804	1.9e-04 [1.1e-04; 5.3e-04]	1.9e-04 [1.3e-04; 2.9e-04]	1.4e-04 [1.1e-04; 2.4e-04]	1.3e-04 [6.7e-05; 2.8e-04]	0.053
3827	3.5e-04 [1.4e-04; 7.2e-04]	1.8e-04 [1.4e-04; 2.7e-04]	1.6e-04 [1.0e-04; 2.5e-04]	8.0e-04 [1.6e-04; 2.1e-03]	<0.001***
3915	1.1e-04 [6.9e-05; 1.5e-04]	1.1e-04 [7.6e-05; 1.2e-04]	8.4e-05 [5.4e-05; 1.3e-04]	1.9e-04 [1.3e-04; 1.9e-04]	0.033*
3934	8.5e-05 [1.7e-05; 1.5e-04]	7.6e-05 [5.6e-05; 1.0e-04]	9.4e-05 [6.6e-05; 1.4e-04]	9.4e-05 [1.1e-05; 1.2e-04]	0.10
3981	2.3e-04 [1.2e-04; 7.0e-04]	4.8e-04 [3.5e-04; 6.1e-04]	3.7e-04 [1.6e-04; 6.8e-04]	6.6e-04 [1.6e-04; 1.9e-03]	0.021*

4138	3.1e-04 [1.6e-04; 8.5e-04]	7.5e-04 [4.1e-04; 1.1e-03]	1.2e-04 [6.9e-05; 2.2e-04]	5.7e-04 [2.2e-04; 1.4e-03]	<0.001***
4160	1.8e-04 [1.4e-04; 2.8e-04]	1.5e-04 [1.2e-04; 2.0e-04]	1.1e-04 [5.9e-05; 1.5e-04]	2.5e-04 [1.9e-04; 3.6e-04]	<0.001***
4192	1.4e-04 [8.5e-05; 1.9e-04]	1.2e-04 [1.1e-04; 1.5e-04]	1.1e-04 [7.1e-05; 1.6e-04]	1.8e-04 [1.5e-04; 4.2e-04]	0.041*
4229	6.1e-05 [1.7e-05; 1.0e-04]	6.7e-05 [4.3e-05; 1.1e-04]	8.2e-05 [4.7e-05; 1.1e-04]	2.6e-05 [5.4e-06; 1.0e-04]	0.086
4356	1.2e-04 [6.0e-05; 1.7e-04]	1.2e-04 [1.1e-04; 1.5e-04]	1.4e-04 [9.8e-05; 2.5e-04]	6.0e-05 [5.2e-05; 8.7e-05]	<0.001***
4374	1.1e-04 [6.6e-05; 1.4e-04]	1.2e-04 [1.0e-04; 1.5e-04]	4.4e-04 [1.3e-04; 2.4e-03]	1.2e-04 [5.9e-05; 1.4e-04]	<0.001***
4393	9.8e-05 [5.0e-05; 1.3e-04]	1.1e-04 [9.3e-05; 1.3e-04]	1.5e-04 [1.1e-04; 2.6e-04]	5.1e-05 [3.6e-05; 9.8e-05]	<0.001***
4428	1.7e-04 [1.2e-04; 2.2e-04]	1.4e-04 [1.2e-04; 1.7e-04]	1.4e-04 [9.8e-05; 1.8e-04]	1.3e-04 [1.1e-04; 1.6e-04]	0.014*
4473	9.5e-05 [4.9e-05; 1.3e-04]	8.9e-05 [4.3e-05; 9.9e-05]	8.6e-05 [5.0e-05; 1.1e-04]	9.2e-05 [8.1e-05; 1.4e-04]	0.3
4532	8.6e-05 [5.2e-05; 1.2e-04]	1.0e-04 [7.4e-05; 1.2e-04]	1.3e-04 [9.7e-05; 2.0e-04]	7.5e-05 [6.6e-05; 1.1e-04]	<0.001***
4551	2.7e-04 [1.6e-04; 4.6e-04]	1.2e-04 [1.1e-04; 1.4e-04]	1.3e-04 [1.0e-04; 1.7e-04]	3.3e-04 [1.3e-04; 4.6e-04]	<0.001***
4574	1.3e-04 [7.8e-05; 1.7e-04]	1.0e-04 [8.1e-05; 1.3e-04]	9.2e-05 [5.0e-05; 1.2e-04]	1.5e-04 [1.1e-04; 1.9e-04]	<0.001***
4636	2.0e-04 [1.2e-04; 3.4e-04]	1.0e-04 [7.4e-05; 1.3e-04]	1.5e-04 [1.2e-04; 2.4e-04]	2.5e-04 [2.1e-04; 3.2e-04]	<0.001***
4715	3.1e-04 [1.3e-04; 8.3e-04]	1.3e-04 [1.0e-04; 1.6e-04]	1.2e-04 [8.4e-05; 1.5e-04]	4.9e-04 [1.7e-04; 9.0e-04]	<0.001***
4738	1.3e-04 [9.8e-05; 1.9e-04]	9.3e-05 [5.7e-05; 1.2e-04]	1.0e-04 [5.2e-05; 1.4e-04]	1.6e-04 [1.2e-04; 1.9e-04]	<0.001***
4811	7.5e-05 [1.7e-05; 1.5e-04]	7.7e-05 [4.2e-05; 1.2e-04]	9.2e-05 [5.9e-05; 1.2e-04]	5.3e-05 [1.1e-05; 1.0e-04]	0.4
4842	2.6e-04 [1.3e-04; 5.9e-04]	1.2e-04 [9.1e-05; 1.5e-04]	8.9e-05 [5.5e-05; 1.2e-04]	2.9e-04 [1.7e-04; 8.7e-04]	<0.001***
4901	1.9e-04 [1.3e-04; 2.9e-04]	7.4e-05 [5.4e-05; 1.0e-04]	9.5e-05 [5.7e-05; 2.0e-04]	8.2e-05 [2.4e-05; 1.5e-04]	<0.001***
4940	1.6e-04 [1.1e-04; 2.6e-04]	3.2e-04 [1.8e-04; 4.2e-04]	2.4e-04 [1.7e-04; 3.7e-04]	1.4e-04 [1.0e-04; 1.8e-04]	<0.001***
4966	2.9e-04 [1.3e-04; 6.4e-04]	3.4e-04 [1.8e-04; 8.5e-04]	2.9e-04 [1.6e-04; 4.6e-04]	4.2e-04 [1.3e-04; 5.2e-04]	0.4
4986	1.3e-04 [7.4e-05; 2.1e-04]	1.2e-04 [8.7e-05; 1.5e-04]	9.5e-05 [3.8e-05; 1.3e-04]	7.2e-05 [5.9e-05; 1.5e-04]	<0.001***
5005	7.1e-05 [1.9e-05; 1.3e-04]	1.1e-04 [6.7e-05; 2.0e-04]	1.0e-04 [7.4e-05; 1.5e-04]	8.4e-05 [8.2e-06; 1.0e-04]	<0.001***
5047	1.3e-04 [7.2e-05; 1.7e-04]	1.2e-04 [8.6e-05; 1.4e-04]	7.3e-05 [3.3e-05; 1.2e-04]	1.1e-04 [7.9e-05; 1.8e-04]	<0.001***
5147	3.8e-04 [1.6e-04; 7.9e-04]	1.3e-04 [1.0e-04; 1.8e-04]	6.8e-05 [3.9e-05; 1.0e-04]	2.1e-04 [1.7e-04; 2.9e-04]	<0.001***
5218	2.5e-04 [1.3e-04; 5.0e-04]	1.7e-04 [1.1e-04; 2.5e-04]	1.7e-04 [1.0e-04; 2.4e-04]	4.3e-04 [1.7e-04; 6.7e-04]	<0.001***
5236	1.7e-03 [2.5e-04; 3.2e-03]	3.4e-04 [2.0e-04; 4.6e-04]	2.4e-04 [1.5e-04; 3.3e-04]	3.9e-03 [1.5e-04; 4.5e-03]	<0.001***
5256	2.3e-04 [1.3e-04; 5.1e-04]	6.7e-05 [1.9e-05; 1.3e-04]	9.8e-05 [6.8e-05; 1.4e-04]	5.3e-04 [1.3e-04; 8.5e-04]	<0.001***
5285	2.4e-04 [1.3e-04; 8.5e-04]	3.7e-04 [2.2e-04; 5.4e-04]	1.7e-04 [9.7e-05; 3.3e-04]	4.7e-04 [1.6e-04; 1.4e-03]	<0.001***
5382	1.9e-03 [2.8e-04; 5.8e-03]	1.5e-03 [9.2e-04; 2.6e-03]	7.1e-04 [1.9e-04; 1.6e-03]	3.2e-03 [1.9e-04; 7.0e-03]	<0.001***
5402	3.8e-04 [1.5e-04; 1.1e-03]	2.0e-04 [1.3e-04; 3.0e-04]	1.6e-04 [9.0e-05; 4.5e-04]	7.9e-04 [1.5e-04; 1.2e-03]	<0.001***
5423	3.9e-04 [1.6e-04; 7.0e-04]	6.4e-05 [3.1e-05; 9.1e-05]	7.9e-05 [4.0e-05; 1.1e-04]	3.3e-04 [1.6e-04; 6.7e-04]	<0.001***
5530	1.8e-04 [9.5e-05; 4.0e-04]	2.1e-04 [1.3e-04; 2.3e-04]	1.7e-04 [1.3e-04; 2.5e-04]	1.6e-04 [1.1e-04; 5.1e-04]	0.9
5594	8.9e-05 [1.6e-05; 1.6e-04]	1.3e-04 [9.3e-05; 2.1e-04]	2.0e-04 [9.3e-05; 9.6e-04]	3.0e-05 [0.0e+00; 3.8e-05]	<0.001***

5869	2.8e-04 [1.2e-04; 4.8e-04]	3.1e-04 [2.1e-04; 3.9e-04]	2.1e-04 [1.3e-04; 3.4e-04]	2.5e-04 [9.3e-05; 3.2e-04]	0.3
5950	1.0e-04 [4.6e-05; 1.4e-04]	2.1e-04 [1.7e-04; 2.7e-04]	3.2e-04 [2.1e-04; 6.2e-04]	1.3e-04 [9.3e-05; 1.3e-04]	<0.001***
6192	1.0e-04 [5.4e-05; 1.5e-04]	1.5e-04 [1.2e-04; 2.1e-04]	1.5e-04 [1.1e-04; 2.0e-04]	9.3e-05 [4.3e-05; 1.2e-04]	<0.001***
6361	1.1e-04 [4.4e-05; 1.9e-04]	7.6e-05 [3.0e-05; 1.1e-04]	1.2e-04 [9.4e-05; 1.5e-04]	1.1e-04 [2.0e-05; 1.5e-04]	<0.001***
6639	8.2e-05 [2.1e-05; 1.4e-04]	9.9e-05 [7.8e-05; 1.3e-04]	1.2e-04 [7.9e-05; 1.6e-04]	3.5e-06 [1.4e-06; 3.5e-05]	<0.001***
6964	5.5e-04 [1.6e-04; 1.5e-03]	2.0e-04 [9.9e-05; 3.4e-04]	1.5e-04 [7.5e-05; 2.6e-04]	1.5e-04 [1.5e-04; 1.9e-03]	<0.001***
7349	4.6e-04 [1.8e-04; 1.1e-03]	1.2e-04 [8.9e-05; 1.7e-04]	1.3e-04 [8.4e-05; 2.1e-04]	1.3e-04 [1.1e-04; 3.9e-04]	<0.001***
7612	2.9e-04 [9.9e-05; 8.2e-04]	7.0e-04 [3.0e-04; 8.3e-04]	3.4e-04 [1.7e-04; 7.3e-04]	1.7e-04 [1.5e-04; 7.1e-04]	0.073
7654	4.5e-05 [1.8e-05; 1.0e-04]	4.5e-05 [2.5e-05; 8.2e-05]	7.6e-05 [3.4e-05; 1.1e-04]	8.8e-05 [2.1e-05; 1.2e-04]	0.010**
7765	2.7e-05 [8.8e-06; 6.6e-05]	8.0e-05 [6.6e-05; 1.1e-04]	1.1e-04 [7.6e-05; 1.8e-04]	1.7e-05 [7.8e-06; 1.2e-04]	<0.001***
8215	1.1e-04 [4.6e-05; 1.7e-04]	9.4e-05 [6.8e-05; 1.4e-04]	9.9e-05 [5.7e-05; 1.3e-04]	7.6e-05 [5.9e-05; 1.1e-04]	0.6
8452	1.0e-04 [2.4e-05; 1.4e-04]	1.1e-04 [7.7e-05; 1.2e-04]	9.5e-05 [7.2e-05; 1.2e-04]	5.5e-05 [2.6e-05; 8.4e-05]	0.2
8469	4.5e-05 [2.2e-05; 9.8e-05]	6.7e-05 [4.3e-05; 1.1e-04]	6.0e-05 [3.6e-05; 9.4e-05]	4.6e-05 [1.5e-05; 1.0e-04]	0.11
8568	1.3e-04 [7.1e-05; 2.1e-04]	7.1e-05 [4.1e-05; 9.4e-05]	6.8e-05 [4.0e-05; 9.4e-05]	1.0e-04 [8.6e-05; 1.3e-04]	<0.001***
8589	7.3e-05 [2.3e-05; 1.3e-04]	9.3e-05 [5.9e-05; 1.1e-04]	7.7e-05 [2.9e-05; 1.1e-04]	2.8e-05 [1.9e-05; 9.0e-05]	0.3
8742	1.3e-04 [3.3e-05; 1.9e-04]	7.7e-05 [5.2e-05; 1.0e-04]	7.9e-05 [5.1e-05; 9.9e-05]	1.4e-04 [1.1e-04; 1.6e-04]	0.001**
9956	5.3e-05 [2.5e-05; 1.1e-04]	6.8e-05 [4.3e-05; 8.8e-05]	9.1e-05 [6.8e-05; 1.1e-04]	5.6e-05 [2.5e-05; 1.2e-04]	<0.001***
10096	1.0e-04 [3.5e-05; 1.5e-04]	7.0e-05 [4.6e-05; 1.1e-04]	8.7e-05 [4.9e-05; 1.1e-04]	3.8e-05 [1.7e-05; 8.5e-05]	0.2
10116	3.4e-05 [1.4e-05; 8.3e-05]	7.2e-05 [5.0e-05; 8.7e-05]	8.3e-05 [4.0e-05; 1.1e-04]	1.3e-05 [1.0e-05; 1.3e-04]	<0.001***
10444	1.7e-04 [1.0e-04; 3.0e-04]	2.0e-04 [1.4e-04; 3.0e-04]	2.1e-04 [1.4e-04; 3.4e-04]	1.4e-04 [1.1e-04; 2.5e-04]	0.059
10837	1.3e-04 [4.6e-05; 1.8e-04]	8.8e-05 [6.0e-05; 1.0e-04]	9.1e-05 [6.0e-05; 1.2e-04]	8.9e-05 [3.6e-05; 1.3e-04]	0.001**
11011	5.1e-05 [1.1e-05; 1.3e-04]	7.3e-05 [4.6e-05; 9.8e-05]	1.0e-04 [7.4e-05; 1.4e-04]	4.8e-05 [1.7e-05; 9.2e-05]	<0.001***
11735	2.3e-04 [1.4e-04; 3.3e-04]	3.2e-04 [2.4e-04; 5.0e-04]	1.6e-04 [9.9e-05; 2.5e-04]	2.0e-04 [8.5e-05; 3.1e-04]	<0.001***
14692	2.8e-04 [1.6e-04; 4.8e-04]	1.1e-04 [8.4e-05; 1.4e-04]	1.2e-04 [9.2e-05; 1.8e-04]	2.2e-04 [1.4e-04; 3.3e-04]	<0.001***

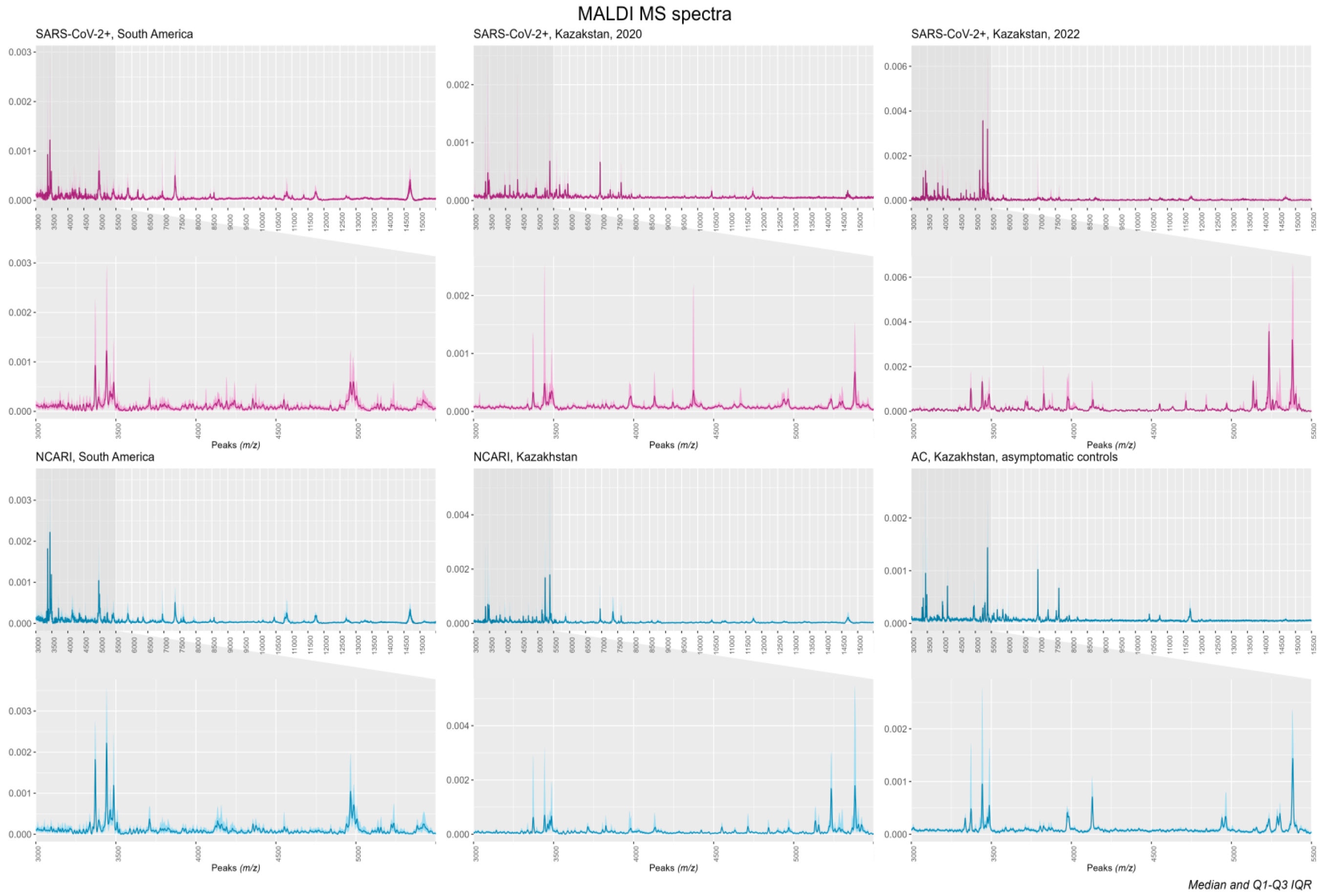

Figure S1. Representative MALDI-MS spectra within each of the participant sub-groups from Kazakhstan (samples collected in the current study) and South America (Nachtigall et al.2020). The central line indicates median value of the spectra, while the shaded region on either side represents the interquartile interval. Insets depict a range from 3000 to 5500 m/z encompassing 70% (62/88) of the identified peaks. NCARI= non-COVID acute respiratory infection. AC= asymptomatic controls.

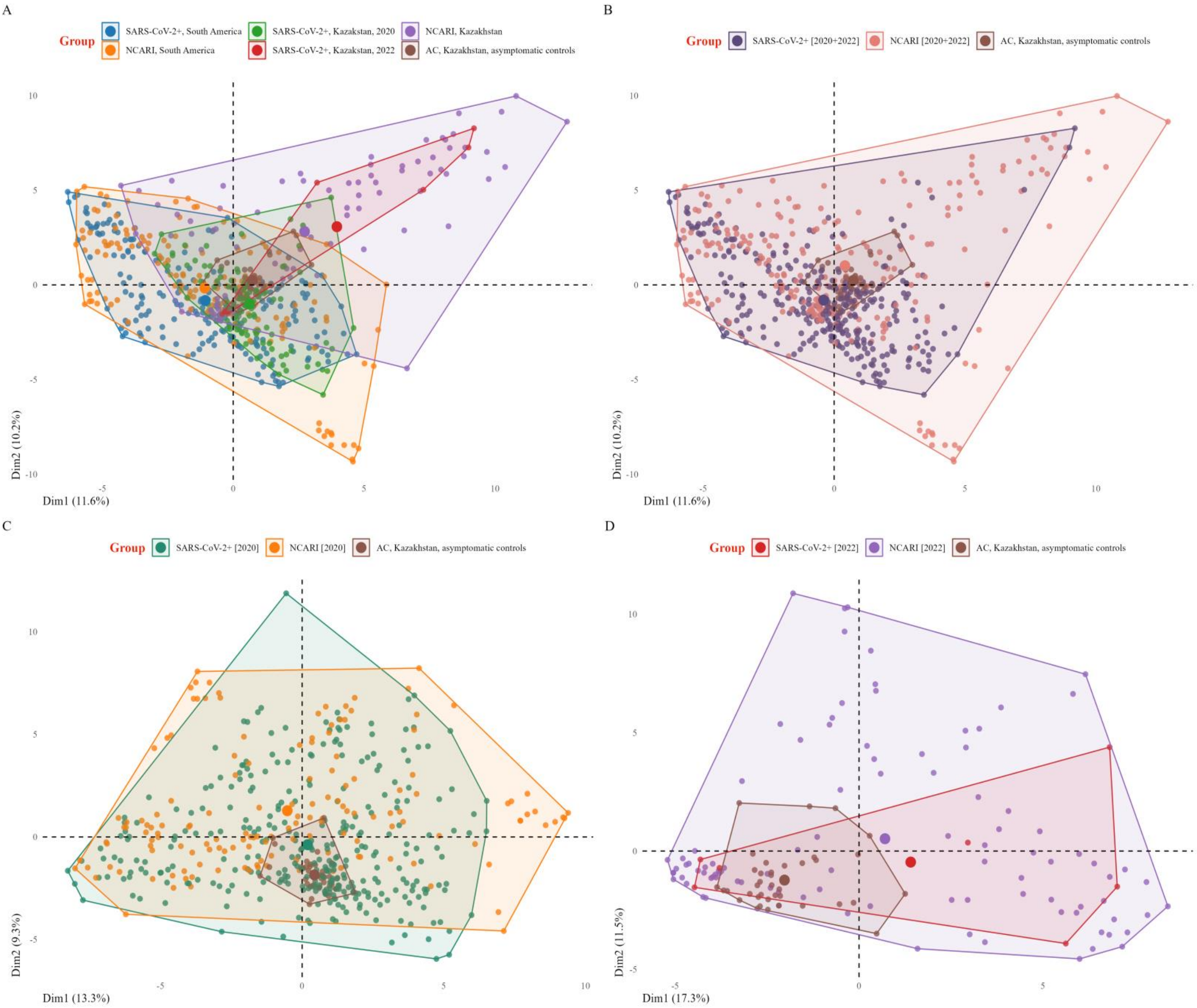

Figure S2. Principal component analysis graphs depicting the first two dimensions of the mass spectra, stratified by sub-group, from the combined dataset based on the peak intensity matrix for Analysis I.

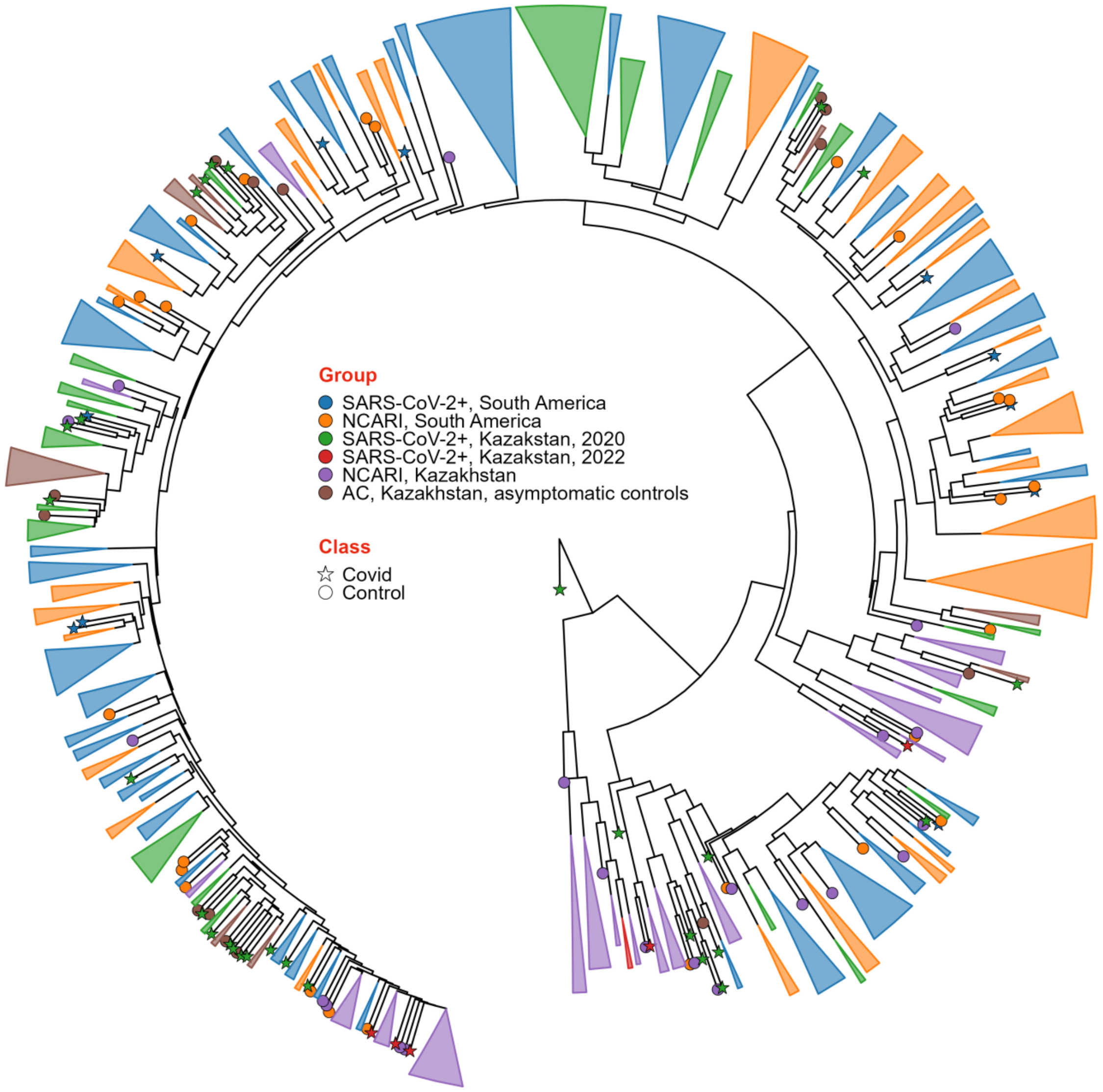

Figure S3. Dendrogram of the mass spectra stratified by sub-group from the combined dataset based on the peak intensity matrix for Analysis I.

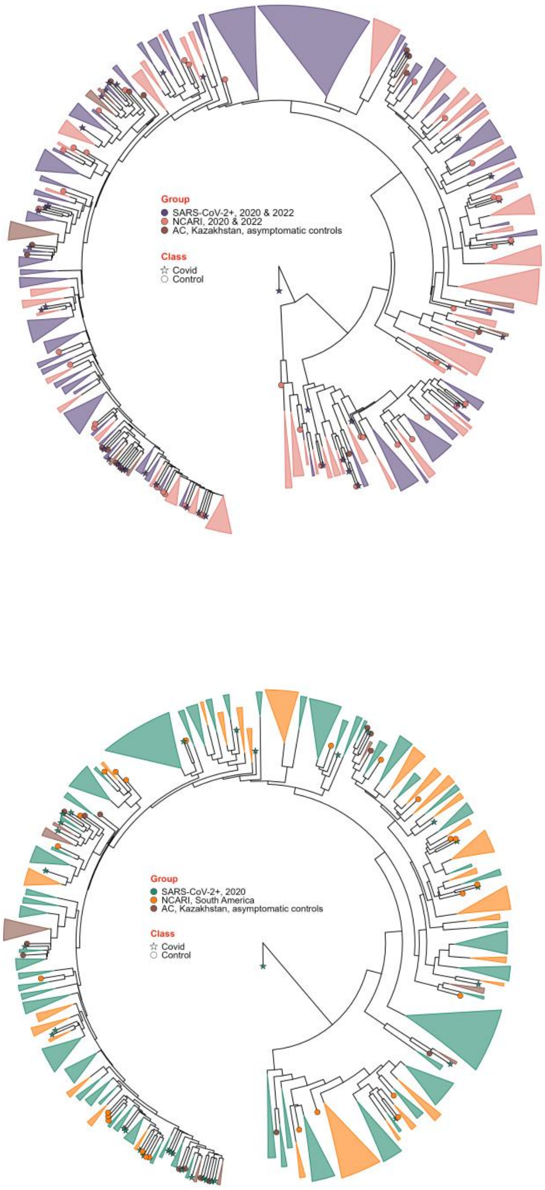

Figure S4. Dendrogram of the mass spectra stratified by sub-group from the combined dataset based on the peak intensity matrix for Analysis I.

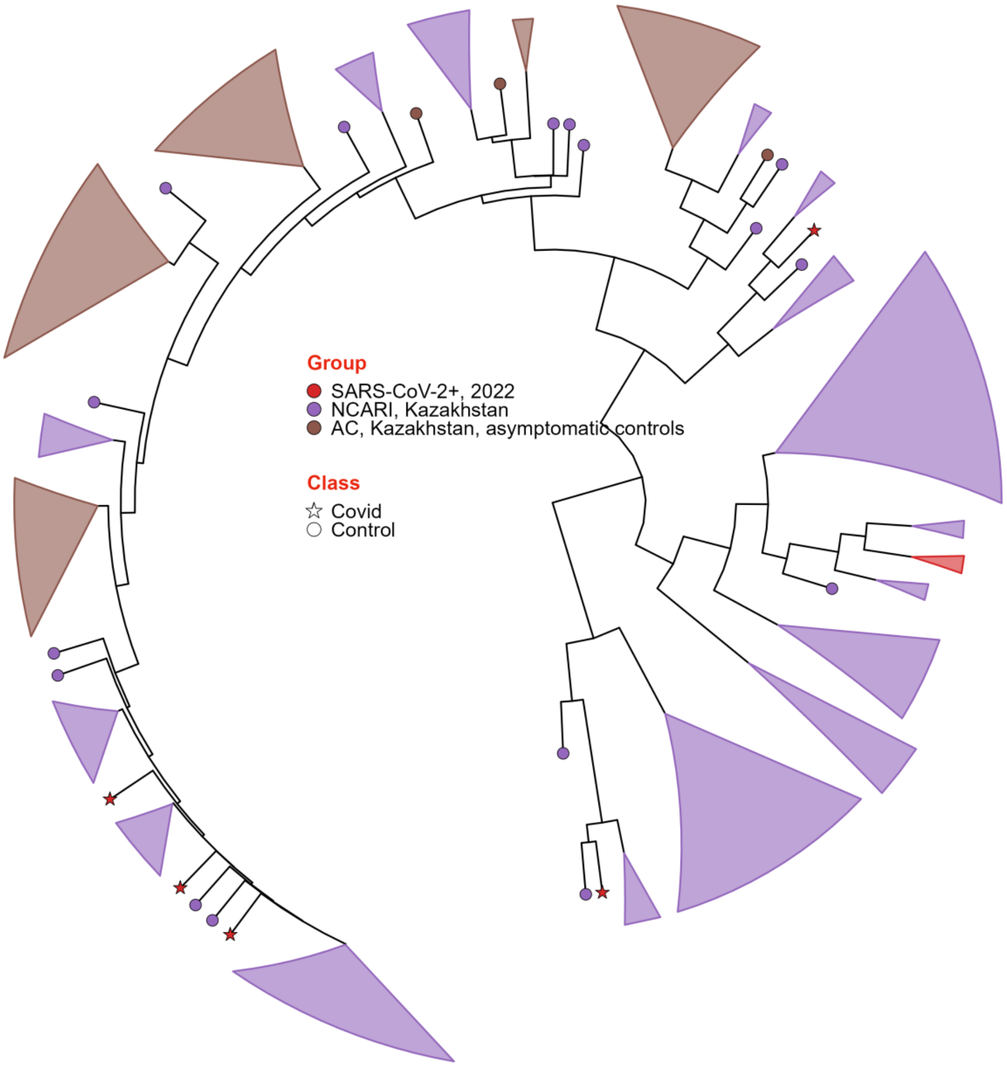

Figure S5. Dendrogram of the mass spectra stratified by sub-group from the combined dataset based on the peak intensity matrix for Analysis I.

Table S3. Performance of seven ML models when tested only on the South American (SA) dataset (Analysis I). This analysis replicated the testing experiment performed by Nachtigall et al. Each metric is presented as a median and interquartile range. Each model was tested 5 times on 20% of the SA dataset.

Classification metric	DT	KNN	NB	RF	SVM-L	SVM-R	XGBoost
**F means**	0.95 [0.94; 0.95]	0.90 [0.88; 0.94]	0.88 [0.86; 0.88]	0.95 [0.93; 0.96]	0.93 [0.91; 0.94]	0.96 [0.95; 0.98]	0.93 [0.93; 0.93]
**Recall**	0.976 [0.953; 0.976]	0.952 [0.951; 0.953]	0.905 [0.857; 0.907]	0.976 [0.976; 0.977]	0.953 [0.951; 0.976]	0.953 [0.952; 0.953]	0.952 [0.907; 0.976]
**Accuracy**	0.94 [0.93; 0.95]	0.88 [0.85; 0.93]	0.85 [0.85; 0.86]	0.94 [0.92; 0.95]	0.92 [0.89; 0.93]	0.96 [0.94; 0.97]	0.92 [0.92; 0.92]
**Specificity**	0.90 [0.87; 0.90]	0.83 [0.68; 0.90]	0.81 [0.80; 0.87]	0.87 [0.83; 0.90]	0.83 [0.81; 0.87]	0.97 [0.97; 1.00]	0.84 [0.83; 0.93]
**Sensitivity**	0.976 [0.953; 0.976]	0.952 [0.951; 0.953]	0.905 [0.857; 0.907]	0.976 [0.976; 0.977]	0.953 [0.951; 0.976]	0.953 [0.952; 0.953]	0.952 [0.907; 0.976]
**PPV**	0.93 [0.91; 0.93]	0.89 [0.80; 0.93]	0.86 [0.86; 0.89]	0.91 [0.89; 0.93]	0.89 [0.87; 0.91]	0.98 [0.97; 1.00]	0.89 [0.89; 0.95]
**NPV**	0.96 [0.94; 0.96]	0.93 [0.89; 0.94]	0.85 [0.81; 0.86]	0.96 [0.96; 0.97]	0.94 [0.92; 0.96]	0.94 [0.93; 0.94]	0.93 [0.88; 0.96]
**ROC AUC**	0.99 [0.99; 0.99]	0.87 [0.83; 0.93]	0.92 [0.91; 0.93]	0.99 [0.97; 1.00]	0.97 [0.97; 0.97]	1.00 [0.99; 1.00]	0.98 [0.98; 0.99]

Table S4. ROC AUC values for comparisons of all Kazakhstan (KZ) SARS-CoV-2+ samples collected in 2020 and 2022 versus the controls (either NCARI or pooled NCARI+AC group, Analysis I). Median [25%; 75%]. Each model was tested 5 times on the complete KZ dataset. NCARI= non-COVID acute respiratory infection. AC=asymptomatic controls.

	**Pairwise comparisons**					
**Model**	**SARS-CoV-2/2020 vs NCARI**	**SARS-CoV-2/2020 vs NCARI+AC**	**SARS-CoV-2/2022 vs NCARI**	**SARS-CoV-2/2022 vs NCARI+AC**	**SARS-CoV-2 [2020 + 2022] vs NCARI**	**SARS-CoV-2 [2020 + 2022] vs NCARI+AC**
**DT**	0.65 [0.65; 0.66]	0.64 [0.63; 0.64]	0.46 [0.42; 0.48]	0.43 [0.38; 0.44]	0.64 [0.64; 0.64]	0.62 [0.62; 0.63]
**KNN**	0.57 [0.55; 0.64]	0.57 [0.55; 0.61]	0.51 [0.47; 0.53]	0.52 [0.48; 0.52]	0.57 [0.54; 0.63]	0.56 [0.55; 0.61]
**NB**	0.68 [0.65; 0.70]	0.66 [0.66; 0.68]	0.52 [0.51; 0.52]	0.51 [0.50; 0.51]	0.67 [0.64; 0.69]	0.65 [0.65; 0.67]
**RF**	0.71 [0.70; 0.73]	0.69 [0.68; 0.70]	0.48 [0.44; 0.48]	0.43 [0.41; 0.44]	0.70 [0.69; 0.71]	0.67 [0.67; 0.68]
**SVM-L**	0.74 [0.72; 0.75]	0.76 [0.72; 0.76]	0.57 [0.56; 0.58]	0.54 [0.52; 0.57]	0.73 [0.71; 0.74]	0.74 [0.71; 0.75]
**SVM-R**	0.60 [0.57; 0.62]	0.60 [0.59; 0.63]	0.58 [0.58; 0.59]	0.61 [0.59; 0.64]	0.60 [0.57; 0.62]	0.60 [0.59; 0.63]
**XG-Boost**	0.65 [0.63; 0.67]	0.64 [0.62; 0.67]	0.30 [0.29; 0.35]	0.29 [0.26; 0.32]	0.62 [0.61; 0.65]	0.62 [0.60; 0.66]

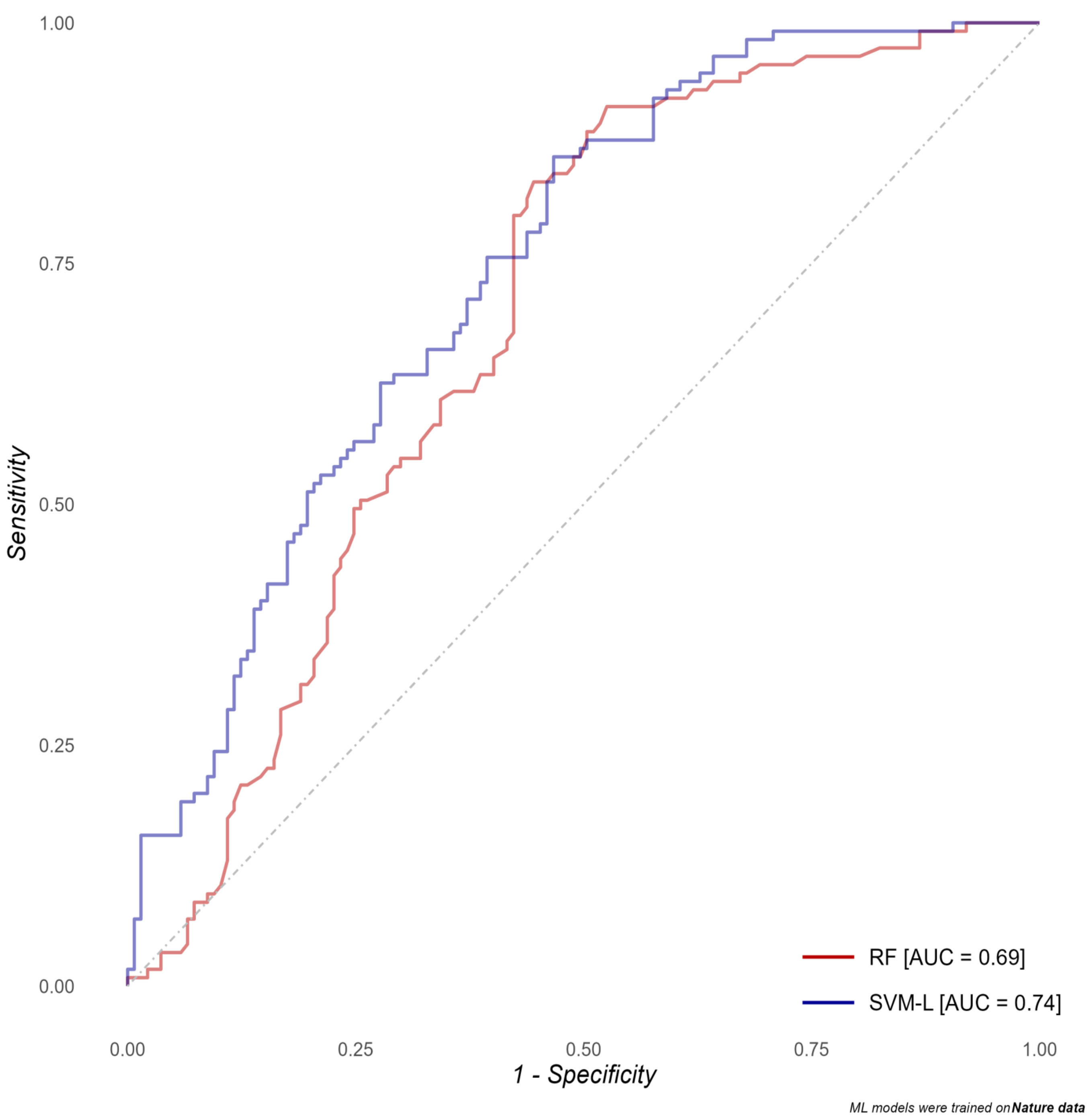

Figure S6. ROC curves for the detection of Kazakhstan SARS-CoV-2 [combined samples from both 2020 and 2022, n=115] vs only NCARI as a control (n=98) for the top two best-performing models (Also see Table S4 for the ROC-AUC values pertaining to each of the seven models). RF=random forest. SVM-L: Support Vector Machine with linear kernel.

Table S5. Mass spectrometry peaks detected across the combined dataset containing both Kazakhstan and South American samples and present in at least 80% of each sub-group. Shown are peak median intensities and interquartile ranges (IQR). p-values were calculated using the two-tailed Wilcoxon rank sum test. NCARI= non-COVID acute respiratory infection. AC=asymptomatic controls.

	South American samples						Kazakhstan samples				
	SARS-CoV-2+			NCARI			NCARI	AC	SARS-CoV-2		
Peak (*m/z*)	Chile N = 10	Argentina N = 167	Peru N = 34	Chile, N = 20	Argentina, N = 114	Peru, N = 17	N = 71	N = 66	2020, N = 108	2023, N = 7	p-value
3013	8.6e-05 [3.4e-05; 1.8e-04]	1.0e-04 [6.2e-05; 1.6e-04]	1.6e-04 [1.1e-04; 2.0e-04]	8.6e-05 [5.3e-05; 1.1e-04]	1.6e-04 [6.6e-05; 3.6e-04]	1.5e-04 [1.1e-04; 1.8e-04]	9.8e-05 [5.3e-05; 1.8e-04]	1.2e-04 [7.8e-05; 2.0e-04]	9.0e-05 [6.0e-05; 1.4e-04]	1.2e-04 [1.1e-04; 1.6e-04]	<0.001***
3037	8.4e-05 [2.3e-05; 1.5e-04]	1.2e-04 [6.7e-05; 2.2e-04]	1.1e-04 [5.8e-05; 1.4e-04]	9.8e-05 [5.7e-05; 1.2e-04]	1.1e-04 [5.1e-05; 2.1e-04]	2.4e-04 [1.5e-04; 4.6e-04]	6.2e-05 [3.4e-05; 1.1e-04]	5.7e-05 [4.1e-05; 9.0e-05]	1.0e-04 [5.5e-05; 3.3e-04]	1.5e-04 [7.7e-05; 1.9e-04]	<0.001***
3052	1.5e-04 [9.9e-05; 2.3e-04]	1.6e-04 [8.9e-05; 2.9e-04]	1.4e-04 [7.3e-05; 2.0e-04]	1.7e-04 [1.5e-04; 2.1e-04]	2.1e-04 [8.9e-05; 4.3e-04]	1.3e-04 [1.0e-04; 2.2e-04]	9.4e-05 [6.4e-05; 1.6e-04]	7.6e-05 [5.5e-05; 1.1e-04]	6.5e-05 [4.3e-05; 1.1e-04]	1.6e-04 [1.2e-04; 3.1e-04]	<0.001***
3095	2.0e-04 [1.6e-04; 2.5e-04]	1.2e-04 [5.5e-05; 2.2e-04]	1.2e-04 [7.0e-05; 1.7e-04]	1.2e-04 [8.2e-05; 1.5e-04]	2.6e-04 [1.5e-04; 5.0e-04]	2.4e-04 [1.5e-04; 2.8e-04]	8.1e-05 [4.5e-05; 1.2e-04]	8.8e-05 [6.7e-05; 1.3e-04]	9.4e-05 [6.6e-05; 1.3e-04]	5.9e-05 [4.0e-05; 1.1e-04]	<0.001***
3113	2.2e-04 [1.3e-04; 3.6e-04]	8.3e-05 [4.0e-05; 1.8e-04]	7.8e-05 [3.3e-05; 1.3e-04]	9.1e-05 [7.1e-05; 1.2e-04]	2.1e-04 [1.3e-04; 2.8e-04]	6.1e-05 [2.7e-05; 9.8e-05]	5.6e-05 [2.1e-05; 9.6e-05]	1.2e-04 [8.3e-05; 1.5e-04]	4.9e-05 [3.1e-05; 8.4e-05]	4.0e-05 [1.3e-05; 4.3e-05]	<0.001***
3128	1.1e-04 [3.7e-05; 1.1e-04]	1.1e-04 [3.8e-05; 2.1e-04]	1.0e-04 [4.3e-05; 1.5e-04]	8.1e-05 [4.3e-05; 1.3e-04]	9.3e-05 [3.8e-05; 1.5e-04]	5.8e-05 [2.4e-05; 9.9e-05]	7.7e-05 [4.2e-05; 1.3e-04]	7.5e-05 [4.2e-05; 1.0e-04]	5.4e-05 [2.9e-05; 7.6e-05]	1.1e-04 [5.6e-05; 2.1e-04]	<0.001***
3147	7.9e-05 [1.9e-05; 1.6e-04]	3.1e-04 [1.5e-04; 5.1e-04]	2.6e-04 [1.7e-04; 3.5e-04]	5.4e-05 [1.8e-05; 1.4e-04]	8.3e-05 [3.1e-05; 1.5e-04]	3.2e-04 [2.2e-04; 3.7e-04]	1.7e-04 [1.3e-04; 2.4e-04]	6.4e-05 [3.8e-05; 9.3e-05]	7.4e-05 [5.0e-05; 1.1e-04]	1.8e-04 [1.4e-04; 2.3e-04]	<0.001***
3192	7.4e-05 [4.0e-05; 1.0e-04]	4.0e-05 [8.8e-06; 1.1e-04]	4.5e-05 [1.5e-05; 1.1e-04]	8.9e-05 [3.1e-05; 1.2e-04]	4.0e-05 [1.1e-06; 1.2e-04]	2.1e-04 [1.5e-04; 3.1e-04]	8.6e-05 [5.4e-05; 1.6e-04]	7.0e-05 [5.2e-05; 1.0e-04]	9.8e-05 [6.5e-05; 1.6e-04]	1.5e-04 [1.2e-04; 1.6e-04]	<0.001***
3218	1.7e-04 [1.0e-04; 2.8e-04]	4.9e-05 [6.5e-06; 1.3e-04]	7.7e-05 [4.7e-05; 1.4e-04]	2.0e-04 [1.3e-04; 2.4e-04]	1.2e-04 [5.2e-05; 1.8e-04]	7.5e-05 [4.3e-05; 1.5e-04]	8.5e-05 [4.7e-05; 1.3e-04]	8.0e-05 [6.8e-05; 9.6e-05]	7.6e-05 [4.3e-05; 9.9e-05]	1.0e-04 [7.4e-05; 1.3e-04]	<0.001***
3242	1.0e-04 [8.8e-05; 1.9e-04]	1.0e-04 [5.2e-05; 1.8e-04]	1.3e-04 [9.3e-05; 1.7e-04]	9.7e-05 [6.9e-05; 1.5e-04]	9.3e-05 [5.0e-05; 1.5e-04]	1.4e-04 [1.1e-04; 1.6e-04]	7.1e-05 [5.0e-05; 1.1e-04]	6.7e-05 [5.3e-05; 1.1e-04]	7.5e-05 [4.9e-05; 1.3e-04]	7.5e-05 [5.6e-05; 1.7e-04]	<0.001***
3256	8.9e-05 [5.5e-05; 1.8e-04]	1.1e-04 [4.0e-05; 2.4e-04]	1.1e-04 [3.5e-05; 1.4e-04]	5.3e-05 [3.2e-05; 1.1e-04]	7.7e-05 [3.1e-05; 1.3e-04]	1.5e-04 [1.1e-04; 1.8e-04]	5.9e-05 [3.4e-05; 8.7e-05]	7.1e-05 [5.5e-05; 1.2e-04]	5.4e-05 [3.7e-05; 8.1e-05]	6.4e-05 [5.7e-05; 9.3e-05]	<0.001***
3300	1.0e-04 [5.7e-05; 1.9e-04]	1.3e-04 [2.4e-05; 3.1e-04]	1.2e-04 [5.8e-05; 2.1e-04]	8.4e-05 [1.7e-05; 1.3e-04]	1.0e-04 [1.1e-05; 3.0e-04]	2.3e-04 [1.6e-04; 3.3e-04]	1.1e-04 [6.7e-05; 2.2e-04]	7.8e-05 [3.9e-05; 1.1e-04]	6.8e-05 [4.2e-05; 8.9e-05]	2.0e-04 [1.9e-04; 3.7e-04]	<0.001***
3318	1.1e-04 [6.5e-05; 1.5e-04]	1.6e-04 [1.0e-07; 3.6e-04]	2.1e-04 [1.5e-04; 3.1e-04]	6.5e-05 [3.0e-06; 2.0e-04]	7.9e-05 [6.7e-06; 3.6e-04]	2.4e-04 [1.8e-04; 3.5e-04]	8.7e-05 [3.8e-05; 1.4e-04]	5.7e-05 [2.0e-05; 1.0e-04]	6.3e-05 [1.9e-05; 9.9e-05]	1.3e-04 [6.5e-05; 1.5e-04]	<0.001***
3337	2.4e-04 [2.0e-04; 3.0e-04]	1.3e-04 [7.2e-06; 3.3e-04]	1.8e-04 [1.5e-04; 2.4e-04]	4.0e-04 [3.2e-04; 6.1e-04]	2.5e-04 [7.5e-05; 5.5e-04]	2.6e-04 [2.1e-04; 4.8e-04]	1.4e-04 [7.2e-05; 4.5e-04]	3.3e-04 [1.6e-04; 4.3e-04]	1.3e-04 [7.8e-05; 2.4e-04]	2.3e-04 [1.8e-04; 4.0e-04]	<0.001***
3358	6.5e-05 [2.2e-06; 9.8e-05]	1.1e-04 [4.0e-05; 1.9e-04]	9.2e-05 [5.3e-05; 1.4e-04]	6.6e-05 [1.7e-05; 1.8e-04]	2.6e-04 [1.9e-04; 3.8e-04]	1.1e-04 [8.6e-05; 1.4e-04]	9.6e-05 [6.2e-05; 1.8e-04]	8.4e-05 [6.3e-05; 1.5e-04]	5.8e-05 [3.0e-05; 1.1e-04]	9.3e-05 [6.2e-05; 1.7e-04]	<0.001***
3372	1.2e-03 [8.6e-04; 2.0e-03]	1.2e-03 [5.7e-04; 3.0e-03]	4.7e-04 [2.9e-04; 6.2e-04]	1.2e-03 [4.4e-04; 2.3e-03]	2.3e-03 [1.2e-03; 3.6e-03]	5.6e-04 [4.2e-04; 6.2e-04]	6.5e-04 [2.4e-04; 3.0e-03]	4.8e-04 [2.5e-04; 1.8e-03]	3.6e-04 [1.8e-04; 1.9e-03]	1.0e-03 [2.0e-04; 1.8e-03]	<0.001***
3393	2.7e-04 [2.0e-04; 5.1e-04]	5.0e-04 [2.5e-04; 8.6e-04]	1.1e-04 [7.8e-05; 1.4e-04]	2.4e-04 [1.5e-04; 3.7e-04]	4.3e-04 [2.6e-04; 7.4e-04]	1.1e-04 [7.7e-05; 1.3e-04]	1.1e-04 [5.1e-05; 2.9e-04]	1.3e-04 [9.3e-05; 2.3e-04]	8.2e-05 [3.5e-05; 1.3e-04]	1.8e-04 [1.1e-04; 2.7e-04]	<0.001***
3443	1.5e-03 [7.5e-04; 2.3e-03]	1.7e-03 [7.5e-04; 4.1e-03]	5.0e-04 [3.1e-04; 6.5e-04]	1.5e-03 [7.3e-04; 2.6e-03]	3.0e-03 [1.5e-03; 4.5e-03]	6.7e-04 [4.7e-04; 8.3e-04]	7.2e-04 [2.2e-04; 3.3e-03]	9.7e-04 [2.2e-04; 2.8e-03]	5.7e-04 [2.7e-04; 2.9e-03]	1.3e-03 [2.3e-04; 1.6e-03]	<0.001***
3463	5.6e-04 [3.7e-04; 7.1e-04]	5.7e-04 [3.0e-04; 1.1e-03]	2.0e-04 [1.5e-04; 2.6e-04]	4.5e-04 [2.7e-04; 6.9e-04]	9.0e-04 [4.2e-04; 1.5e-03]	3.0e-04 [2.1e-04; 4.3e-04]	3.9e-04 [2.0e-04; 8.3e-04]	1.6e-04 [1.2e-04; 2.5e-04]	1.9e-04 [1.3e-04; 5.7e-04]	4.7e-04 [1.0e-04; 8.0e-04]	<0.001***
3476	2.7e-04 [2.1e-04; 3.2e-04]	3.9e-04 [2.2e-04; 6.0e-04]	2.3e-04 [1.7e-04; 3.2e-04]	3.9e-04 [2.5e-04; 6.2e-04]	3.9e-04 [2.4e-04; 6.8e-04]	3.2e-04 [2.1e-04; 4.6e-04]	5.2e-04 [1.4e-04; 8.7e-04]	3.0e-04 [2.1e-04; 4.9e-04]	3.3e-04 [1.5e-04; 5.1e-04]	2.7e-04 [1.2e-04; 9.2e-04]	0.001**
3487	1.1e-03 [7.8e-04; 1.7e-03]	7.5e-04 [3.0e-04; 2.0e-03]	2.8e-04 [1.6e-04; 5.1e-04]	9.4e-04 [3.2e-04; 2.2e-03]	1.5e-03 [7.4e-04; 3.4e-03]	3.5e-04 [3.0e-04; 6.4e-04]	7.3e-04 [2.9e-04; 2.0e-03]	5.7e-04 [1.8e-04; 1.8e-03]	3.4e-04 [1.4e-04; 1.1e-03]	7.8e-04 [2.0e-04; 1.6e-03]	<0.001***
3516	2.1e-04 [1.2e-04; 4.6e-04]	4.4e-05 [0.0e+00; 1.7e-04]	1.1e-04 [5.2e-05; 1.4e-04]	5.7e-05 [6.5e-06; 1.3e-04]	1.9e-05 [0.0e+00; 3.7e-04]	2.1e-04 [1.5e-04; 2.7e-04]	8.5e-05 [4.1e-05; 1.5e-04]	7.0e-05 [3.6e-05; 1.0e-04]	6.6e-05 [3.8e-05; 1.0e-04]	1.2e-04 [6.9e-05; 1.7e-04]	<0.001***
3560	1.6e-05 [4.9e-06; 3.4e-05]	1.6e-06 [0.0e+00; 5.8e-05]	7.1e-05 [3.0e-05; 1.2e-04]	3.3e-05 [6.4e-06; 1.0e-04]	2.1e-05 [0.0e+00; 1.2e-04]	1.1e-04 [2.5e-05; 1.7e-04]	1.1e-04 [7.1e-05; 1.5e-04]	5.9e-05 [4.7e-05; 7.2e-05]	5.2e-05 [1.9e-05; 9.0e-05]	1.7e-04 [1.2e-04; 2.2e-04]	<0.001***
3589	1.3e-04 [6.2e-05; 1.7e-04]	7.9e-05 [2.1e-05; 1.6e-04]	8.1e-05 [6.2e-05; 1.2e-04]	1.4e-04 [8.8e-05; 2.2e-04]	1.2e-04 [5.0e-05; 2.2e-04]	1.7e-04 [8.0e-05; 2.7e-04]	1.3e-04 [8.4e-05; 2.5e-04]	1.6e-04 [1.2e-04; 2.1e-04]	9.0e-05 [4.9e-05; 1.4e-04]	2.1e-04 [1.6e-04; 4.0e-04]	<0.001***
3610	7.2e-05 [5.8e-05; 1.4e-04]	1.1e-04 [3.8e-05; 1.8e-04]	8.7e-05 [3.8e-05; 1.5e-04]	1.4e-04 [6.1e-05; 2.3e-04]	1.0e-04 [5.0e-05; 2.0e-04]	1.3e-04 [1.2e-04; 1.8e-04]	1.2e-04 [6.6e-05; 1.7e-04]	6.7e-05 [4.6e-05; 8.9e-05]	6.1e-05 [2.8e-05; 9.1e-05]	2.2e-04 [1.7e-04; 3.4e-04]	<0.001***
3633	1.1e-04 [7.8e-05; 1.5e-04]	6.9e-05 [1.5e-05; 1.3e-04]	1.1e-04 [4.8e-05; 2.1e-04]	1.2e-04 [5.3e-05; 1.8e-04]	1.7e-04 [8.3e-05; 2.8e-04]	1.1e-04 [6.2e-05; 1.4e-04]	1.3e-04 [8.7e-05; 2.6e-04]	1.3e-04 [1.0e-04; 1.7e-04]	9.2e-05 [6.2e-05; 1.3e-04]	1.7e-04 [1.2e-04; 3.2e-04]	<0.001***

3658	1.3e-04 [1.0e-04; 1.4e-04]	2.3e-05 [3.5e-06; 7.4e-05]	6.2e-05 [2.1e-05; 1.1e-04]	4.0e-05 [4.5e-07; 1.0e-04]	6.5e-05 [1.5e-05; 1.4e-04]	7.5e-05 [4.9e-05; 1.1e-04]	4.5e-05 [2.1e-05; 8.3e-05]	6.8e-05 [2.9e-05; 8.7e-05]	7.0e-05 [5.1e-05; 1.1e-04]	1.6e-04 [1.5e-04; 2.7e-04]	<0.001***
3711	2.4e-04 [1.2e-04; 4.8e-04]	3.3e-04 [1.3e-04; 7.4e-04]	1.4e-04 [6.5e-05; 2.3e-04]	3.1e-04 [2.1e-04; 5.5e-04]	4.2e-04 [2.2e-04; 8.7e-04]	1.1e-04 [6.1e-05; 2.4e-04]	2.2e-04 [1.0e-04; 4.2e-04]	1.1e-04 [8.2e-05; 2.0e-04]	1.0e-04 [6.2e-05; 1.9e-04]	4.6e-04 [1.8e-04; 6.3e-04]	<0.001***
3732	1.4e-04 [1.2e-04; 3.4e-04]	8.8e-05 [3.4e-05; 1.7e-04]	8.0e-05 [4.7e-05; 1.0e-04]	1.1e-04 [4.1e-05; 1.8e-04]	1.1e-04 [2.2e-05; 2.0e-04]	6.0e-05 [1.5e-05; 9.6e-05]	9.0e-05 [4.5e-05; 1.4e-04]	9.8e-05 [7.2e-05; 1.7e-04]	9.1e-05 [6.0e-05; 1.3e-04]	8.8e-05 [8.0e-05; 2.2e-04]	0.034*
3752	6.2e-05 [4.5e-06; 8.9e-05]	1.2e-04 [2.1e-05; 2.5e-04]	1.7e-04 [8.9e-05; 3.4e-04]	8.5e-05 [4.3e-05; 1.3e-04]	8.2e-05 [4.1e-06; 1.6e-04]	2.6e-04 [1.5e-04; 3.7e-04]	8.7e-05 [5.3e-05; 1.4e-04]	8.1e-05 [5.6e-05; 1.2e-04]	1.0e-04 [6.0e-05; 1.7e-04]	2.1e-04 [1.2e-04; 2.4e-04]	<0.001***
3776	1.1e-04 [5.0e-05; 2.3e-04]	1.3e-04 [7.0e-05; 2.6e-04]	1.3e-04 [7.8e-05; 2.6e-04]	1.2e-04 [8.0e-05; 1.8e-04]	1.2e-04 [5.6e-05; 2.5e-04]	2.1e-04 [1.6e-04; 3.6e-04]	4.4e-05 [2.0e-05; 7.8e-05]	7.4e-05 [4.5e-05; 9.0e-05]	8.3e-05 [4.9e-05; 1.4e-04]	1.3e-04 [1.1e-04; 2.1e-04]	<0.001***
3792	2.8e-04 [1.2e-04; 1.1e-03]	1.6e-04 [5.2e-05; 3.5e-04]	6.7e-05 [2.6e-05; 9.0e-05]	2.2e-04 [1.3e-04; 5.7e-04]	1.6e-04 [6.5e-05; 3.7e-04]	5.7e-05 [1.3e-05; 8.0e-05]	3.5e-05 [9.2e-06; 8.0e-05]	6.2e-05 [4.0e-05; 8.3e-05]	5.3e-05 [2.6e-05; 7.7e-05]	1.4e-05 [4.8e-06; 5.0e-05]	<0.001***
3805	1.4e-04 [1.2e-04; 1.5e-04]	1.4e-04 [2.3e-05; 2.6e-04]	2.5e-04 [1.8e-04; 4.2e-04]	2.3e-04 [1.9e-04; 3.0e-04]	9.5e-05 [2.2e-05; 3.0e-04]	2.6e-04 [1.3e-04; 4.9e-04]	1.7e-04 [8.4e-05; 4.4e-04]	1.9e-04 [1.2e-04; 2.9e-04]	1.2e-04 [7.7e-05; 2.3e-04]	1.7e-04 [1.3e-04; 2.8e-04]	<0.001***
3827	7.7e-05 [3.2e-05; 1.1e-04]	1.2e-04 [4.4e-05; 2.7e-04]	1.8e-04 [1.0e-04; 3.4e-04]	9.9e-05 [5.2e-05; 2.4e-04]	1.5e-04 [5.5e-05; 3.6e-04]	3.8e-04 [1.6e-04; 5.8e-04]	3.5e-04 [1.4e-04; 7.2e-04]	1.7e-04 [1.3e-04; 2.6e-04]	1.3e-04 [6.5e-05; 2.1e-04]	8.0e-04 [1.5e-04; 2.1e-03]	<0.001***
3848	3.7e-05 [1.7e-05; 5.8e-05]	5.6e-05 [1.3e-05; 1.3e-04]	8.2e-05 [3.0e-05; 1.5e-04]	9.7e-06 [0.0e+00; 6.2e-05]	9.1e-05 [3.4e-05; 1.7e-04]	9.7e-05 [5.7e-05; 2.2e-04]	8.7e-05 [5.1e-05; 1.5e-04]	6.5e-05 [5.4e-05; 1.0e-04]	4.6e-05 [1.7e-05; 8.6e-05]	1.4e-04 [1.0e-04; 2.9e-04]	<0.001***
3862	3.2e-05 [1.9e-06; 5.8e-05]	3.3e-05 [7.6e-07; 7.2e-05]	4.8e-05 [1.7e-05; 1.2e-04]	5.6e-05 [3.4e-05; 1.4e-04]	7.8e-05 [1.6e-05; 1.3e-04]	9.8e-05 [6.7e-05; 1.6e-04]	1.3e-04 [8.4e-05; 3.5e-04]	1.2e-04 [8.8e-05; 2.0e-04]	9.0e-05 [4.6e-05; 1.5e-04]	2.7e-04 [1.4e-04; 6.0e-04]	<0.001***
3877	4.1e-05 [2.5e-05; 9.0e-05]	5.5e-05 [2.3e-05; 1.2e-04]	2.9e-05 [4.8e-06; 8.4e-05]	7.9e-05 [5.4e-05; 1.4e-04]	6.9e-05 [2.0e-05; 1.3e-04]	8.6e-05 [4.4e-05; 1.3e-04]	8.5e-05 [4.2e-05; 1.2e-04]	1.5e-04 [9.7e-05; 2.5e-04]	1.1e-04 [7.2e-05; 1.9e-04]	2.1e-04 [2.0e-04; 8.9e-04]	<0.001***
3901	2.6e-05 [9.3e-06; 4.4e-05]	2.6e-05 [2.7e-06; 7.1e-05]	4.2e-05 [1.5e-05; 6.1e-05]	7.0e-05 [1.6e-05; 9.3e-05]	1.5e-05 [0.0e+00; 6.9e-05]	1.9e-05 [7.4e-06; 4.1e-05]	4.5e-05 [1.3e-05; 1.0e-04]	7.6e-05 [4.4e-05; 9.8e-05]	5.4e-05 [1.9e-05; 7.6e-05]	1.7e-04 [1.0e-04; 1.9e-04]	<0.001***
3917	1.5e-04 [7.9e-05; 2.0e-04]	1.2e-04 [5.3e-05; 1.8e-04]	9.1e-05 [4.8e-05; 1.5e-04]	2.2e-04 [1.5e-04; 2.7e-04]	1.6e-04 [8.9e-05; 2.4e-04]	9.6e-05 [3.7e-05; 1.2e-04]	7.5e-05 [4.6e-05; 1.2e-04]	7.4e-05 [5.1e-05; 1.0e-04]	7.9e-05 [5.0e-05; 1.1e-04]	1.9e-04 [1.5e-04; 1.9e-04]	<0.001***
3934	1.8e-04 [1.4e-04; 3.2e-04]	1.4e-04 [5.6e-05; 2.3e-04]	1.6e-04 [1.2e-04; 2.2e-04]	1.1e-04 [6.9e-05; 1.6e-04]	1.4e-04 [4.4e-05; 2.8e-04]	1.6e-04 [8.5e-05; 2.3e-04]	4.0e-05 [6.6e-06; 1.0e-04]	7.3e-05 [5.6e-05; 9.8e-05]	9.4e-05 [6.6e-05; 1.4e-04]	1.2e-05 [1.0e-05; 7.6e-05]	<0.001***
3980	6.8e-05 [1.6e-05; 1.1e-04]	1.9e-04 [1.2e-04; 2.7e-04]	1.7e-04 [1.1e-04; 2.7e-04]	1.4e-05 [1.0e-07; 1.9e-04]	1.1e-04 [5.2e-05; 1.8e-04]	3.0e-04 [1.4e-04; 4.8e-04]	2.0e-04 [8.8e-05; 5.8e-04]	4.4e-04 [2.9e-04; 5.9e-04]	2.5e-04 [1.1e-04; 4.8e-04]	6.6e-04 [1.3e-04; 1.8e-03]	<0.001***
4000	1.0e-04 [4.2e-05; 1.8e-04]	5.9e-05 [2.9e-05; 1.1e-04]	4.8e-05 [2.5e-05; 1.0e-04]	5.4e-05 [5.5e-06; 1.2e-04]	5.9e-05 [2.5e-05; 1.0e-04]	1.2e-04 [6.8e-05; 3.2e-04]	8.0e-05 [4.8e-05; 1.3e-04]	1.0e-04 [7.6e-05; 1.3e-04]	8.4e-05 [4.7e-05; 1.3e-04]	1.8e-04 [1.3e-04; 2.8e-04]	<0.001***
4136	2.7e-04 [1.9e-04; 5.4e-04]	1.9e-04 [1.1e-04; 4.2e-04]	1.3e-04 [6.5e-05; 2.0e-04]	1.1e-04 [5.9e-05; 2.7e-04]	4.7e-04 [2.2e-04; 9.3e-04]	2.1e-04 [1.3e-04; 3.3e-04]	2.5e-04 [1.1e-04; 4.7e-04]	7.5e-04 [4.1e-04; 1.1e-03]	1.5e-04 [7.2e-05; 3.1e-04]	5.7e-04 [2.2e-04; 1.4e-03]	<0.001***
4157	2.3e-04 [1.8e-04; 4.1e-04]	1.4e-04 [6.8e-05; 3.2e-04]	8.3e-05 [2.2e-05; 1.3e-04]	1.4e-04 [3.5e-05; 2.4e-04]	3.6e-04 [1.0e-04; 9.0e-04]	1.6e-04 [5.6e-05; 2.4e-04]	1.1e-04 [6.0e-05; 1.9e-04]	1.0e-04 [8.1e-05; 1.3e-04]	6.2e-05 [3.0e-05; 1.0e-04]	2.5e-04 [1.9e-04; 3.6e-04]	<0.001***
4171	9.9e-05 [6.2e-05; 1.5e-04]	5.1e-05 [8.2e-06; 1.1e-04]	6.7e-05 [3.6e-05; 1.2e-04]	8.6e-05 [4.8e-05; 1.2e-04]	1.3e-04 [3.3e-05; 4.0e-04]	9.7e-05 [5.0e-05; 2.0e-04]	1.2e-04 [6.9e-05; 2.2e-04]	1.2e-04 [9.7e-05; 1.7e-04]	7.5e-05 [4.0e-05; 1.2e-04]	1.9e-04 [1.6e-04; 3.4e-04]	<0.001***
4193	6.2e-05 [1.8e-05; 8.0e-05]	1.4e-04 [2.2e-05; 5.8e-04]	6.4e-04 [3.8e-04; 8.5e-04]	7.3e-05 [2.4e-05; 1.7e-04]	1.7e-04 [5.5e-05; 3.5e-04]	3.7e-04 [2.6e-04; 5.8e-04]	8.7e-05 [5.1e-05; 1.3e-04]	9.2e-05 [6.7e-05; 1.2e-04]	8.5e-05 [5.7e-05; 1.4e-04]	1.8e-04 [1.5e-04; 4.2e-04]	<0.001***
4229	3.7e-04 [1.7e-04; 6.8e-04]	1.5e-04 [8.7e-05; 2.9e-04]	1.3e-04 [6.6e-05; 2.1e-04]	2.9e-04 [2.0e-04; 5.9e-04]	1.3e-04 [4.8e-05; 3.1e-04]	7.8e-05 [2.6e-05; 1.0e-04]	4.8e-05 [1.6e-05; 8.8e-05]	6.4e-05 [4.3e-05; 8.2e-05]	6.6e-05 [4.0e-05; 1.0e-04]	2.6e-05 [5.4e-06; 4.3e-05]	<0.001***
4305	1.4e-05 [6.3e-07; 4.2e-05]	3.2e-05 [8.1e-06; 6.9e-05]	6.4e-05 [1.2e-05; 9.1e-05]	4.0e-05 [1.6e-05; 1.0e-04]	2.0e-05 [1.2e-06; 1.0e-04]	1.7e-04 [1.2e-05; 2.8e-04]	7.7e-05 [4.1e-05; 1.1e-04]	8.3e-05 [5.4e-05; 1.1e-04]	5.9e-05 [2.5e-05; 8.7e-05]	7.7e-05 [6.7e-05; 1.1e-04]	<0.001***
4356	1.8e-04 [1.3e-04; 2.2e-04]	3.0e-04 [1.9e-04; 4.3e-04]	2.3e-04 [1.9e-04; 2.8e-04]	1.8e-04 [4.9e-05; 2.2e-04]	3.4e-04 [2.1e-04; 4.5e-04]	1.8e-04 [1.5e-04; 2.3e-04]	7.0e-05 [3.6e-05; 1.2e-04]	8.8e-05 [6.1e-05; 1.2e-04]	1.2e-04 [7.6e-05; 2.3e-04]	5.9e-05 [3.9e-05; 5.9e-05]	<0.001***
4374	1.6e-04 [1.4e-04; 4.2e-04]	1.8e-04 [1.2e-04; 5.7e-04]	3.6e-04 [1.3e-04; 6.8e-04]	2.3e-04 [1.4e-04; 3.9e-04]	1.9e-04 [8.9e-05; 3.5e-04]	6.2e-04 [2.3e-04; 1.0e-03]	7.7e-05 [4.9e-05; 1.0e-04]	8.7e-05 [6.4e-05; 1.3e-04]	4.4e-04 [1.2e-04; 2.3e-03]	4.7e-05 [3.8e-05; 6.0e-05]	<0.001***
4393	1.8e-04 [1.7e-04; 2.2e-04]	9.5e-05 [4.7e-05; 2.4e-04]	1.9e-04 [1.4e-04; 3.3e-04]	1.6e-04 [1.1e-04; 2.6e-04]	1.4e-04 [8.3e-05; 2.3e-04]	2.9e-04 [4.4e-05; 5.3e-04]	6.2e-05 [3.6e-05; 9.8e-05]	8.6e-05 [6.2e-05; 1.0e-04]	1.1e-04 [6.8e-05; 2.0e-04]	4.6e-05 [3.6e-05; 6.5e-05]	<0.001***
4428	1.4e-04 [1.1e-04; 2.2e-04]	6.0e-05 [1.7e-05; 1.3e-04]	7.3e-05 [4.0e-05; 9.8e-05]	6.1e-05 [7.4e-06; 1.1e-04]	6.3e-05 [1.8e-05; 1.5e-04]	2.9e-04 [2.0e-04; 3.5e-04]	7.7e-05 [4.7e-05; 1.2e-04]	8.7e-05 [5.8e-05; 1.2e-04]	9.7e-05 [6.6e-05; 1.4e-04]	7.4e-05 [4.1e-05; 8.4e-05]	<0.001***
4471	1.7e-04 [1.6e-04; 1.9e-04]	9.2e-05 [4.3e-05; 1.4e-04]	1.2e-04 [6.1e-05; 1.8e-04]	1.0e-04 [6.8e-05; 1.2e-04]	1.1e-04 [4.8e-05; 2.0e-04]	1.1e-04 [3.1e-05; 1.3e-04]	8.6e-05 [4.7e-05; 1.1e-04]	6.7e-05 [4.0e-05; 9.2e-05]	6.7e-05 [3.6e-05; 9.4e-05]	9.2e-05 [8.1e-05; 1.4e-04]	<0.001***
4532	5.9e-05 [3.1e-05; 1.6e-04]	1.2e-04 [8.3e-05; 1.8e-04]	2.0e-04 [1.6e-04; 2.6e-04]	7.7e-05 [1.4e-05; 1.2e-04]	3.8e-05 [1.2e-05; 9.6e-05]	2.5e-04 [1.5e-04; 3.7e-04]	5.7e-05 [2.9e-05; 9.1e-05]	7.5e-05 [5.9e-05; 1.0e-04]	1.1e-04 [7.2e-05; 1.7e-04]	7.4e-05 [6.6e-05; 8.2e-05]	<0.001***

4551	2.5e-04 [6.6e-05; 2.9e-04]	2.1e-04 [8.2e-05; 3.2e-04]	5.2e-04 [2.6e-04; 8.0e-04]	3.2e-04 [2.1e-04; 5.6e-04]	1.3e-04 [6.1e-05; 3.7e-04]	5.9e-04 [4.8e-04; 6.6e-04]	2.7e-04 [1.6e-04; 4.6e-04]	8.5e-05 [7.0e-05; 1.1e-04]	9.4e-05 [6.7e-05; 1.3e-04]	3.3e-04 [1.3e-04; 4.6e-04]	<0.001***
4574	1.6e-04 [1.3e-04; 2.2e-04]	1.4e-04 [4.2e-05; 2.3e-04]	1.5e-04 [9.6e-05; 2.5e-04]	1.4e-04 [9.6e-05; 2.6e-04]	9.6e-05 [3.5e-05; 1.5e-04]	1.7e-04 [1.4e-04; 2.3e-04]	8.6e-05 [5.1e-05; 1.2e-04]	6.7e-05 [5.4e-05; 1.0e-04]	5.1e-05 [3.5e-05; 8.4e-05]	1.5e-04 [1.1e-04; 1.9e-04]	<0.001***
4635	1.3e-04 [7.2e-05; 2.4e-04]	1.2e-04 [7.5e-05; 1.8e-04]	1.7e-04 [1.0e-04; 3.5e-04]	1.8e-04 [1.3e-04; 2.4e-04]	7.9e-05 [4.3e-05; 1.2e-04]	3.0e-04 [1.5e-04; 4.3e-04]	1.4e-04 [8.6e-05; 2.5e-04]	9.6e-05 [7.8e-05; 1.2e-04]	1.1e-04 [7.9e-05; 1.4e-04]	2.5e-04 [2.1e-04; 3.2e-04]	<0.001***
4714	2.0e-04 [1.6e-04; 4.3e-04]	1.1e-04 [5.7e-05; 1.8e-04]	1.4e-04 [8.2e-05; 2.9e-04]	2.3e-04 [7.7e-05; 5.1e-04]	8.6e-05 [4.0e-05; 1.5e-04]	1.3e-04 [7.9e-05; 4.5e-04]	2.9e-04 [1.2e-04; 8.1e-04]	1.0e-04 [8.2e-05; 1.3e-04]	8.4e-05 [4.8e-05; 1.1e-04]	4.9e-04 [1.7e-04; 9.0e-04]	<0.001***
4738	1.7e-04 [1.2e-04; 3.1e-04]	6.0e-05 [2.0e-05; 1.4e-04]	3.7e-05 [1.3e-05; 1.0e-04]	2.5e-04 [1.3e-04; 2.6e-04]	4.1e-05 [1.0e-05; 1.0e-04]	9.6e-05 [7.4e-05; 1.2e-04]	9.8e-05 [5.8e-05; 1.5e-04]	6.7e-05 [5.2e-05; 9.8e-05]	5.9e-05 [2.6e-05; 9.6e-05]	1.1e-04 [7.1e-05; 1.2e-04]	<0.001***
4763	8.9e-05 [3.1e-05; 2.0e-04]	7.5e-05 [3.8e-05; 1.2e-04]	5.6e-05 [3.5e-05; 9.2e-05]	1.2e-04 [3.9e-05; 1.7e-04]	9.6e-05 [2.3e-05; 1.9e-04]	4.3e-05 [3.2e-05; 1.1e-04]	7.6e-05 [3.7e-05; 1.2e-04]	5.2e-05 [3.7e-05; 8.2e-05]	5.1e-05 [2.2e-05; 8.4e-05]	1.4e-04 [9.7e-05; 1.6e-04]	<0.001***
4811	1.4e-04 [8.5e-05; 1.7e-04]	4.6e-05 [1.7e-05; 8.0e-05]	8.3e-05 [4.9e-05; 1.1e-04]	5.3e-05 [2.8e-05; 9.9e-05]	4.8e-05 [1.4e-05; 1.3e-04]	7.1e-05 [4.0e-05; 1.2e-04]	4.7e-05 [1.5e-05; 1.1e-04]	6.2e-05 [3.4e-05; 8.5e-05]	6.6e-05 [4.4e-05; 9.4e-05]	3.9e-05 [1.1e-05; 9.5e-05]	<0.001***
4842	1.8e-04 [1.1e-04; 3.0e-04]	1.0e-04 [5.3e-05; 2.3e-04]	1.5e-04 [6.7e-05; 2.4e-04]	2.0e-04 [1.3e-04; 3.1e-04]	9.9e-05 [4.2e-05; 2.4e-04]	1.9e-04 [1.8e-04; 2.3e-04]	2.6e-04 [1.2e-04; 5.9e-04]	1.2e-04 [9.1e-05; 1.5e-04]	9.1e-05 [5.3e-05; 1.3e-04]	2.9e-04 [1.7e-04; 8.7e-04]	<0.001***
4901	1.8e-04 [7.3e-05; 2.8e-04]	9.9e-05 [4.3e-05; 1.9e-04]	3.3e-04 [1.8e-04; 5.1e-04]	2.0e-04 [1.3e-04; 2.6e-04]	5.4e-05 [2.1e-05; 1.2e-04]	2.8e-04 [2.0e-04; 4.4e-04]	1.3e-04 [7.3e-05; 2.6e-04]	6.9e-05 [5.2e-05; 9.5e-05]	9.2e-05 [5.3e-05; 1.9e-04]	4.4e-05 [1.7e-05; 8.9e-05]	<0.001***
4941	2.7e-04 [1.2e-04; 8.4e-04]	1.9e-04 [1.3e-04; 3.8e-04]	2.4e-04 [1.5e-04; 3.5e-04]	3.1e-04 [1.8e-04; 4.8e-04]	2.5e-04 [1.1e-04; 4.9e-04]	2.6e-04 [1.3e-04; 3.0e-04]	1.2e-04 [6.5e-05; 2.3e-04]	3.2e-04 [1.8e-04; 4.2e-04]	2.2e-04 [1.4e-04; 3.4e-04]	1.2e-04 [8.6e-05; 1.7e-04]	<0.001***
4966	1.4e-03 [3.7e-04; 2.4e-03]	6.8e-04 [3.8e-04; 1.4e-03]	5.1e-04 [4.1e-04; 6.9e-04]	1.3e-03 [9.5e-04; 1.8e-03]	1.2e-03 [6.4e-04; 2.4e-03]	3.6e-04 [2.5e-04; 8.0e-04]	1.9e-04 [7.2e-05; 5.3e-04]	3.4e-04 [1.4e-04; 8.5e-04]	2.0e-04 [9.8e-05; 4.0e-04]	4.2e-04 [1.3e-04; 5.2e-04]	<0.001***
4986	9.7e-04 [6.2e-04; 1.5e-03]	8.7e-04 [4.7e-04; 1.4e-03]	3.1e-04 [2.3e-04; 3.7e-04]	9.1e-04 [6.0e-04; 1.1e-03]	9.2e-04 [5.1e-04; 1.6e-03]	3.4e-04 [1.6e-04; 4.2e-04]	1.1e-04 [5.0e-05; 2.0e-04]	8.6e-05 [6.9e-05; 1.4e-04]	4.7e-05 [2.2e-05; 9.5e-05]	7.1e-05 [5.9e-05; 1.2e-04]	<0.001***
5005	3.5e-04 [2.1e-04; 6.6e-04]	4.7e-04 [2.0e-04; 7.5e-04]	1.7e-04 [1.2e-04; 2.3e-04]	3.7e-04 [2.8e-04; 4.5e-04]	5.2e-04 [2.4e-04; 9.0e-04]	1.9e-04 [3.4e-05; 2.5e-04]	4.5e-05 [1.8e-05; 9.1e-05]	9.4e-05 [4.1e-05; 1.7e-04]	7.1e-05 [3.3e-05; 1.0e-04]	1.6e-05 [2.1e-07; 7.9e-05]	<0.001***
5043	1.5e-04 [1.2e-04; 2.9e-04]	8.0e-05 [2.8e-05; 1.6e-04]	5.2e-05 [2.4e-05; 8.2e-05]	4.4e-05 [9.3e-06; 1.6e-04]	7.8e-05 [2.8e-05; 2.1e-04]	2.3e-05 [1.1e-05; 6.4e-05]	6.7e-05 [2.6e-05; 1.0e-04]	7.3e-05 [5.4e-05; 1.0e-04]	5.9e-05 [2.3e-05; 8.8e-05]	1.1e-04 [7.9e-05; 1.8e-04]	<0.001***
5059	5.0e-05 [3.5e-05; 8.4e-05]	4.0e-05 [9.2e-06; 7.2e-05]	6.3e-05 [3.6e-05; 1.2e-04]	7.4e-05 [4.0e-05; 1.6e-04]	5.4e-05 [5.5e-06; 1.9e-04]	6.2e-05 [9.3e-06; 1.3e-04]	9.4e-05 [5.7e-05; 1.4e-04]	5.6e-05 [2.6e-05; 8.5e-05]	5.6e-05 [3.0e-05; 8.4e-05]	1.4e-04 [1.2e-04; 3.1e-04]	<0.001***
5136	1.6e-04 [4.7e-05; 2.6e-04]	1.4e-04 [4.2e-05; 4.1e-04]	2.9e-04 [1.6e-04; 3.7e-04]	1.9e-04 [1.1e-04; 2.6e-04]	8.5e-05 [2.2e-05; 3.4e-04]	2.3e-04 [1.6e-04; 3.9e-04]	5.4e-04 [1.6e-04; 1.2e-03]	8.9e-05 [6.6e-05; 1.2e-04]	7.4e-05 [4.7e-05; 1.1e-04]	1.4e-03 [1.3e-04; 1.6e-03]	<0.001***
5156	1.2e-04 [7.7e-05; 2.4e-04]	1.6e-04 [4.4e-05; 3.3e-04]	1.6e-04 [6.2e-05; 2.9e-04]	3.2e-04 [1.6e-04; 4.0e-04]	1.2e-04 [5.4e-05; 4.6e-04]	3.0e-04 [1.9e-04; 4.2e-04]	3.8e-04 [1.5e-04; 7.9e-04]	1.3e-04 [8.3e-05; 1.6e-04]	9.2e-05 [7.0e-05; 1.3e-04]	6.4e-04 [1.7e-04; 1.4e-03]	<0.001***
5177	2.9e-05 [1.4e-05; 9.7e-05]	8.2e-05 [4.0e-05; 1.6e-04]	8.5e-05 [5.0e-05; 1.3e-04]	9.2e-05 [4.4e-05; 1.4e-04]	3.7e-05 [1.1e-05; 2.0e-04]	1.2e-04 [6.7e-05; 1.7e-04]	7.1e-05 [4.0e-05; 1.3e-04]	2.6e-05 [4.8e-06; 6.0e-05]	6.1e-05 [3.1e-05; 1.0e-04]	1.4e-04 [1.3e-04; 1.7e-04]	<0.001***
5191	2.8e-06 [0.0e+00; 6.2e-05]	2.6e-05 [4.9e-06; 6.8e-05]	6.2e-05 [3.2e-05; 9.0e-05]	3.0e-05 [2.1e-06; 8.3e-05]	2.0e-05 [9.0e-07; 5.6e-05]	5.1e-05 [3.1e-05; 6.7e-05]	7.6e-05 [2.9e-05; 1.0e-04]	4.9e-05 [3.3e-05; 7.1e-05]	5.9e-05 [2.9e-05; 9.3e-05]	1.1e-04 [7.7e-05; 1.2e-04]	<0.001***
5218	7.7e-05 [3.7e-05; 1.3e-04]	9.9e-05 [4.1e-05; 2.7e-04]	2.7e-04 [1.6e-04; 3.3e-04]	1.7e-04 [9.7e-05; 2.0e-04]	1.7e-04 [7.6e-05; 3.2e-04]	1.6e-04 [7.6e-05; 2.7e-04]	2.2e-04 [1.0e-04; 4.3e-04]	1.3e-04 [7.8e-05; 1.8e-04]	1.2e-04 [7.3e-05; 1.7e-04]	3.9e-04 [1.4e-04; 6.7e-04]	<0.001***
5235	1.3e-04 [6.4e-05; 4.9e-04]	1.0e-04 [3.8e-05; 5.9e-04]	3.2e-04 [1.7e-04; 6.3e-04]	5.3e-04 [2.1e-04; 9.9e-04]	2.1e-04 [6.8e-05; 6.1e-04]	4.2e-04 [3.0e-04; 5.9e-04]	1.7e-03 [2.5e-04; 3.2e-03]	3.4e-04 [2.0e-04; 4.6e-04]	2.1e-04 [1.3e-04; 3.1e-04]	3.9e-03 [1.5e-04; 4.5e-03]	<0.001***
5256	6.2e-05 [2.6e-05; 1.9e-04]	7.4e-05 [2.3e-05; 2.2e-04]	1.4e-04 [5.4e-05; 2.3e-04]	1.8e-04 [8.5e-05; 2.9e-04]	7.6e-05 [2.0e-05; 2.8e-04]	1.8e-04 [1.3e-04; 2.1e-04]	1.7e-04 [8.0e-05; 4.3e-04]	5.1e-05 [1.4e-05; 8.4e-05]	6.3e-05 [2.9e-05; 9.1e-05]	5.3e-04 [1.3e-04; 8.5e-04]	<0.001***
5286	9.8e-05 [5.5e-05; 1.2e-04]	3.7e-05 [7.8e-06; 9.1e-05]	1.0e-04 [5.7e-05; 2.6e-04]	7.6e-05 [4.0e-05; 1.5e-04]	2.3e-05 [2.3e-09; 9.6e-05]	1.8e-04 [1.5e-04; 3.0e-04]	2.3e-04 [1.1e-04; 7.9e-04]	3.7e-04 [2.2e-04; 5.4e-04]	1.6e-04 [7.1e-05; 3.2e-04]	4.7e-04 [1.6e-04; 1.4e-03]	<0.001***
5303	4.5e-05 [8.7e-06; 9.2e-05]	5.0e-05 [2.1e-05; 9.7e-05]	9.3e-05 [5.1e-05; 1.6e-04]	6.9e-05 [1.4e-05; 1.3e-04]	3.2e-05 [4.9e-06; 1.3e-04]	1.8e-04 [8.8e-05; 3.0e-04]	1.7e-04 [8.0e-05; 9.3e-04]	4.3e-04 [2.6e-04; 6.6e-04]	1.7e-04 [6.4e-05; 4.1e-04]	2.4e-04 [1.8e-04; 2.0e-03]	<0.001***
5382	1.8e-04 [1.0e-04; 4.9e-04]	1.3e-04 [5.4e-05; 2.3e-04]	4.5e-04 [2.1e-04; 7.6e-04]	3.8e-04 [2.1e-04; 5.7e-04]	1.7e-04 [9.4e-05; 2.7e-04]	8.7e-04 [6.3e-04; 1.0e-03]	1.9e-03 [2.8e-04; 5.8e-03]	1.5e-03 [9.2e-04; 2.6e-03]	7.0e-04 [1.9e-04; 1.6e-03]	3.2e-03 [1.9e-04; 7.0e-03]	<0.001***
5403	2.0e-04 [8.5e-05; 2.9e-04]	1.3e-04 [6.8e-05; 2.5e-04]	2.3e-04 [8.4e-05; 3.4e-04]	1.8e-04 [1.3e-04; 2.1e-04]	1.2e-04 [5.3e-05; 2.3e-04]	3.7e-04 [2.5e-04; 4.8e-04]	3.3e-04 [9.1e-05; 9.5e-04]	1.6e-04 [1.0e-04; 2.8e-04]	1.3e-04 [5.0e-05; 3.5e-04]	7.9e-04 [1.5e-04; 1.2e-03]	<0.001***
5423	3.7e-04 [1.9e-04; 6.1e-04]	2.1e-04 [1.0e-04; 4.6e-04]	2.0e-04 [1.4e-04; 2.8e-04]	3.6e-04 [2.0e-04; 8.4e-04]	2.7e-04 [8.7e-05; 5.4e-04]	2.1e-04 [1.7e-04; 3.3e-04]	3.6e-04 [1.3e-04; 6.0e-04]	6.4e-05 [3.3e-05; 9.1e-05]	7.9e-05 [4.0e-05; 1.1e-04]	3.3e-04 [1.5e-04; 6.7e-04]	<0.001***
5510	1.7e-05 [4.8e-06; 3.4e-05]	6.0e-05 [1.6e-05; 1.2e-04]	1.1e-04 [7.3e-05; 1.8e-04]	3.4e-05 [4.1e-06; 9.5e-05]	2.2e-05 [3.9e-06; 6.1e-05]	1.2e-04 [3.1e-05; 1.8e-04]	5.0e-05 [1.1e-05; 8.2e-05]	5.5e-05 [3.0e-05; 7.3e-05]	5.5e-05 [2.5e-05; 8.7e-05]	1.1e-04 [7.2e-05; 1.4e-04]	<0.001***

5529	3.7e-05 [3.7e-07; 1.1e-04]	5.1e-05 [4.9e-06; 1.4e-04]	1.5e-04 [7.6e-05; 2.3e-04]	6.4e-05 [2.7e-05; 1.6e-04]	5.2e-05 [9.9e-06; 1.5e-04]	2.0e-04 [1.3e-04; 3.4e-04]	1.4e-04 [6.9e-05; 3.1e-04]	1.8e-04 [1.3e-04; 2.2e-04]	1.3e-04 [9.4e-05; 1.9e-04]	1.6e-04 [1.1e-04; 5.1e-04]	<0.001***
5594	8.6e-05 [5.0e-05; 1.9e-04]	8.6e-05 [4.5e-05; 3.2e-04]	2.0e-04 [9.3e-05; 4.6e-04]	1.5e-04 [9.6e-05; 2.4e-04]	6.6e-05 [2.3e-05; 1.6e-04]	2.3e-04 [1.7e-04; 5.3e-04]	5.0e-05 [1.3e-05; 1.3e-04]	7.4e-05 [5.3e-05; 1.6e-04]	1.9e-04 [7.9e-05; 8.8e-04]	3.0e-05 [0.0e+00; 3.8e-05]	<0.001***
5687	1.3e-04 [4.7e-05; 2.1e-04]	1.2e-04 [5.6e-05; 2.1e-04]	2.2e-04 [1.2e-04; 4.3e-04]	8.9e-05 [7.2e-05; 1.7e-04]	7.7e-05 [3.1e-05; 1.5e-04]	1.1e-04 [4.9e-05; 2.5e-04]	9.2e-05 [5.2e-05; 1.6e-04]	3.1e-04 [2.1e-04; 4.2e-04]	3.2e-04 [1.8e-04; 4.8e-04]	9.7e-05 [6.2e-05; 1.2e-04]	<0.001***
5763	5.0e-05 [1.9e-05; 8.0e-05]	3.7e-05 [5.9e-06; 8.1e-05]	7.9e-05 [4.6e-05; 1.3e-04]	6.5e-05 [4.4e-05; 1.0e-04]	4.4e-05 [9.8e-06; 9.3e-05]	5.6e-05 [2.3e-05; 8.9e-05]	7.0e-05 [3.3e-05; 1.6e-04]	1.5e-04 [1.1e-04; 2.2e-04]	9.1e-05 [5.4e-05; 1.6e-04]	4.1e-05 [6.7e-06; 9.8e-05]	<0.001***
5864	3.4e-04 [1.5e-04; 5.2e-04]	1.9e-04 [1.0e-04; 5.7e-04]	5.0e-04 [3.5e-04; 6.6e-04]	3.8e-04 [2.9e-04; 5.2e-04]	1.3e-04 [7.5e-05; 3.7e-04]	4.8e-04 [2.2e-04; 5.6e-04]	1.7e-04 [6.3e-05; 3.4e-04]	3.1e-04 [2.1e-04; 3.9e-04]	1.9e-04 [1.0e-04; 3.0e-04]	1.3e-04 [6.6e-05; 3.0e-04]	<0.001***
5948	5.9e-05 [5.1e-05; 2.1e-04]	6.9e-05 [3.0e-05; 1.3e-04]	1.9e-04 [1.0e-04; 3.1e-04]	6.3e-05 [3.6e-05; 8.1e-05]	4.5e-05 [8.9e-06; 8.2e-05]	2.3e-04 [1.3e-04; 3.2e-04]	6.8e-05 [3.8e-05; 1.1e-04]	1.6e-04 [1.1e-04; 2.1e-04]	2.6e-04 [1.6e-04; 5.0e-04]	9.0e-05 [6.7e-05; 1.2e-04]	<0.001***
5971	6.6e-05 [6.8e-06; 2.3e-04]	4.8e-05 [3.3e-06; 1.4e-04]	1.1e-04 [6.0e-05; 1.5e-04]	6.4e-05 [9.0e-06; 3.0e-04]	1.7e-05 [4.4e-07; 6.8e-05]	9.9e-05 [8.3e-05; 2.2e-04]	4.4e-05 [1.1e-05; 1.1e-04]	1.5e-04 [8.3e-05; 2.0e-04]	9.5e-05 [5.2e-05; 1.4e-04]	1.1e-04 [8.5e-05; 2.5e-04]	<0.001***
6192	2.1e-04 [1.1e-04; 2.5e-04]	2.3e-04 [9.8e-05; 4.1e-04]	1.8e-04 [1.3e-04; 2.8e-04]	1.4e-04 [1.3e-04; 1.8e-04]	1.8e-04 [1.2e-04; 2.5e-04]	1.2e-04 [5.3e-05; 1.4e-04]	8.1e-05 [4.7e-05; 1.2e-04]	1.1e-04 [7.8e-05; 1.5e-04]	9.6e-05 [5.9e-05; 1.4e-04]	6.5e-05 [2.3e-05; 9.1e-05]	<0.001***
6227	4.2e-05 [2.2e-05; 9.4e-05]	3.1e-05 [1.1e-05; 7.5e-05]	5.9e-05 [3.8e-05; 9.0e-05]	4.2e-05 [2.1e-05; 9.0e-05]	3.4e-05 [8.3e-06; 7.3e-05]	5.1e-05 [1.8e-05; 8.4e-05]	2.8e-05 [7.4e-06; 5.2e-05]	7.9e-05 [5.2e-05; 1.0e-04]	6.5e-05 [3.7e-05; 1.0e-04]	7.9e-05 [6.0e-05; 1.3e-04]	<0.001***
6278	6.0e-05 [2.8e-05; 8.8e-05]	3.7e-05 [9.5e-06; 6.5e-05]	6.5e-05 [2.8e-05; 9.5e-05]	4.6e-05 [1.3e-05; 7.1e-05]	2.3e-05 [5.9e-06; 4.7e-05]	8.3e-05 [6.4e-05; 1.0e-04]	3.6e-05 [1.9e-05; 8.3e-05]	1.3e-04 [9.9e-05; 1.7e-04]	8.2e-05 [5.6e-05; 1.1e-04]	2.7e-05 [1.5e-05; 4.8e-05]	<0.001***
6361	2.2e-04 [1.7e-04; 2.5e-04]	8.9e-05 [3.4e-05; 1.6e-04]	6.5e-05 [2.5e-05; 1.1e-04]	1.4e-04 [5.1e-05; 2.7e-04]	1.4e-04 [4.7e-05; 2.4e-04]	8.4e-05 [1.7e-05; 1.2e-04]	6.5e-05 [2.1e-05; 1.5e-04]	5.7e-05 [2.6e-05; 7.3e-05]	6.9e-05 [4.7e-05; 1.0e-04]	1.6e-05 [4.3e-06; 8.7e-05]	<0.001***
6639	7.1e-05 [3.3e-05; 1.0e-04]	8.2e-05 [4.7e-05; 1.2e-04]	2.3e-04 [1.5e-04; 3.5e-04]	1.3e-04 [1.1e-04; 1.5e-04]	7.1e-05 [3.9e-05; 1.6e-04]	9.5e-05 [6.0e-05; 2.0e-04]	3.8e-05 [1.3e-05; 1.0e-04]	6.2e-05 [4.6e-05; 9.7e-05]	8.4e-05 [6.2e-05; 1.4e-04]	3.5e-06 [1.4e-06; 3.1e-05]	<0.001***
6953	4.5e-04 [1.7e-04; 6.5e-04]	1.4e-04 [3.4e-05; 8.2e-04]	2.8e-04 [1.2e-04; 8.4e-04]	4.5e-04 [1.8e-04; 1.5e-03]	1.9e-04 [5.2e-05; 7.3e-04]	1.4e-04 [7.0e-05; 1.2e-03]	5.5e-04 [1.5e-04; 1.5e-03]	1.2e-03 [4.9e-04; 1.6e-03]	7.2e-04 [2.9e-04; 1.4e-03]	1.5e-04 [1.5e-04; 1.9e-03]	<0.001***
6974	2.5e-04 [1.6e-04; 4.4e-04]	8.3e-05 [2.4e-05; 2.9e-04]	1.4e-04 [9.5e-05; 3.3e-04]	1.9e-04 [9.3e-05; 6.3e-04]	9.8e-05 [4.2e-05; 4.4e-04]	1.9e-04 [1.1e-04; 6.4e-04]	1.5e-04 [7.2e-05; 4.1e-04]	1.2e-04 [7.7e-05; 1.9e-04]	9.6e-05 [6.4e-05; 1.6e-04]	1.1e-04 [7.4e-05; 3.4e-04]	<0.001***
7093	3.6e-05 [1.3e-05; 7.0e-05]	4.0e-05 [1.1e-05; 7.9e-05]	7.0e-05 [3.9e-05; 9.8e-05]	8.5e-05 [3.6e-05; 1.5e-04]	2.3e-05 [5.6e-06; 4.4e-05]	4.8e-05 [2.5e-05; 5.7e-05]	7.9e-05 [3.3e-05; 1.3e-04]	7.2e-05 [4.6e-05; 1.0e-04]	9.9e-05 [7.2e-05; 1.4e-04]	8.3e-05 [4.1e-05; 1.3e-04]	<0.001***
7207	2.2e-05 [1.4e-05; 3.5e-05]	2.5e-05 [3.8e-06; 5.3e-05]	2.2e-05 [4.5e-06; 5.2e-05]	5.7e-05 [3.3e-06; 8.7e-05]	7.8e-06 [1.2e-07; 2.8e-05]	1.5e-05 [4.3e-06; 4.9e-05]	1.4e-05 [1.9e-06; 5.1e-05]	1.4e-04 [1.0e-04; 1.9e-04]	1.1e-04 [6.5e-05; 1.7e-04]	2.5e-05 [1.2e-05; 2.9e-05]	<0.001***
7270	1.3e-05 [0.0e+00; 3.1e-05]	6.1e-05 [2.1e-05; 1.1e-04]	9.4e-05 [1.9e-05; 1.6e-04]	2.9e-05 [0.0e+00; 6.2e-05]	2.1e-05 [3.0e-08; 5.7e-05]	6.5e-05 [3.0e-05; 1.1e-04]	5.6e-05 [2.3e-05; 1.1e-04]	2.8e-04 [1.5e-04; 5.6e-04]	2.1e-04 [1.1e-04; 3.7e-04]	5.0e-05 [1.6e-05; 1.8e-04]	<0.001***
7349	1.3e-03 [8.0e-04; 1.7e-03]	4.8e-04 [2.2e-04; 1.0e-03]	6.7e-04 [4.1e-04; 1.0e-03]	8.5e-04 [6.9e-04; 1.1e-03]	4.6e-04 [1.8e-04; 8.5e-04]	5.5e-04 [2.5e-04; 7.6e-04]	4.1e-04 [1.3e-04; 1.0e-03]	7.3e-05 [4.7e-05; 1.6e-04]	1.1e-04 [5.7e-05; 1.7e-04]	1.2e-04 [1.0e-04; 3.9e-04]	<0.001***
7531	9.6e-05 [4.8e-05; 1.5e-04]	4.4e-05 [3.0e-06; 1.6e-04]	9.3e-05 [3.6e-05; 2.2e-04]	1.6e-04 [7.8e-05; 3.2e-04]	8.8e-05 [2.6e-05; 1.8e-04]	1.3e-04 [7.8e-05; 2.4e-04]	9.4e-05 [3.5e-05; 2.1e-04]	2.5e-04 [1.7e-04; 3.4e-04]	1.4e-04 [8.7e-05; 2.2e-04]	8.5e-05 [4.2e-05; 2.3e-04]	<0.001***
7611	9.8e-05 [7.3e-05; 1.9e-04]	5.8e-05 [1.8e-05; 1.5e-04]	2.1e-04 [9.8e-05; 6.9e-04]	3.1e-04 [2.5e-04; 6.7e-04]	9.1e-05 [3.5e-05; 3.5e-04]	6.0e-04 [1.5e-04; 1.1e-03]	2.8e-04 [8.1e-05; 7.3e-04]	7.0e-04 [3.0e-04; 8.3e-04]	3.4e-04 [1.7e-04; 7.3e-04]	1.6e-04 [1.1e-04; 7.1e-04]	<0.001***
7654	2.1e-05 [0.0e+00; 7.0e-05]	3.0e-05 [9.3e-06; 6.1e-05]	8.2e-05 [4.7e-05; 1.2e-04]	6.9e-05 [2.8e-05; 1.3e-04]	4.1e-05 [1.5e-05; 8.9e-05]	1.6e-04 [1.1e-04; 2.4e-04]	4.3e-05 [1.8e-05; 7.2e-05]	3.4e-05 [2.3e-05; 5.5e-05]	4.5e-05 [3.1e-05; 7.9e-05]	2.3e-05 [1.8e-05; 8.0e-05]	<0.001***
7765	2.1e-05 [6.7e-06; 4.2e-05]	2.8e-05 [5.9e-06; 6.6e-05]	9.8e-05 [5.3e-05; 1.5e-04]	5.4e-05 [1.3e-05; 1.0e-04]	1.9e-05 [3.7e-06; 3.7e-05]	1.3e-04 [1.0e-04; 3.4e-04]	2.1e-05 [7.9e-06; 5.3e-05]	4.5e-05 [2.9e-05; 6.9e-05]	5.6e-05 [2.7e-05; 9.3e-05]	1.6e-05 [7.8e-06; 2.8e-05]	<0.001***
7932	1.9e-04 [1.1e-04; 2.0e-04]	4.8e-05 [1.5e-05; 8.9e-05]	6.9e-05 [3.6e-05; 1.1e-04]	1.5e-04 [5.3e-05; 2.3e-04]	5.8e-05 [2.6e-05; 2.1e-04]	7.9e-05 [1.1e-05; 1.1e-04]	4.2e-05 [1.7e-05; 6.9e-05]	1.8e-04 [1.2e-04; 2.8e-04]	1.2e-04 [8.0e-05; 2.1e-04]	4.1e-05 [2.5e-05; 8.2e-05]	<0.001***
8215	1.3e-04 [1.1e-04; 1.9e-04]	5.5e-05 [2.7e-05; 1.0e-04]	6.9e-05 [4.4e-05; 1.0e-04]	7.0e-05 [5.0e-05; 1.1e-04]	2.8e-05 [1.1e-05; 6.7e-05]	1.8e-04 [3.3e-05; 6.6e-04]	6.7e-05 [3.6e-05; 1.3e-04]	7.9e-05 [6.2e-05; 1.1e-04]	7.2e-05 [5.2e-05; 1.0e-04]	6.2e-05 [4.5e-05; 7.6e-05]	<0.001***
8452	2.5e-04 [1.4e-04; 7.2e-04]	1.5e-04 [7.5e-05; 2.6e-04]	8.1e-05 [5.6e-05; 1.2e-04]	2.9e-04 [1.5e-04; 5.2e-04]	9.3e-05 [5.4e-05; 1.5e-04]	5.1e-05 [2.7e-05; 7.2e-05]	5.8e-05 [2.2e-05; 1.1e-04]	6.9e-05 [5.7e-05; 1.0e-04]	6.6e-05 [4.0e-05; 9.4e-05]	4.1e-05 [1.9e-05; 4.9e-05]	<0.001***
8469	1.8e-04 [1.3e-04; 3.9e-04]	6.1e-05 [2.4e-05; 1.1e-04]	5.4e-05 [1.8e-05; 8.4e-05]	1.2e-04 [5.0e-05; 2.0e-04]	4.2e-05 [2.2e-05; 8.4e-05]	5.3e-05 [2.7e-05; 7.4e-05]	4.4e-05 [2.2e-05; 9.2e-05]	6.1e-05 [4.2e-05; 8.1e-05]	5.4e-05 [3.4e-05; 7.2e-05]	2.2e-05 [1.5e-05; 5.6e-05]	<0.001***
8568	1.0e-04 [7.6e-05; 1.3e-04]	1.4e-04 [6.4e-05; 2.3e-04]	3.1e-04 [2.6e-04; 4.9e-04]	1.5e-04 [6.9e-05; 3.1e-04]	1.6e-04 [9.6e-05; 3.1e-04]	2.4e-04 [1.4e-04; 4.1e-04]	1.1e-04 [5.3e-05; 1.8e-04]	4.4e-05 [3.4e-05; 6.7e-05]	6.4e-05 [4.0e-05; 8.3e-05]	1.5e-04 [1.0e-04; 1.5e-04]	<0.001***
8589	3.5e-05 [1.0e-05; 4.3e-05]	4.0e-05 [6.7e-06; 8.6e-05]	1.4e-04 [1.1e-04; 1.7e-04]	1.1e-04 [5.6e-05; 1.8e-04]	4.6e-05 [1.7e-05; 7.8e-05]	9.7e-05 [5.5e-05; 1.5e-04]	4.8e-05 [2.1e-05; 8.3e-05]	4.0e-05 [2.4e-05; 6.6e-05]	4.3e-05 [2.4e-05; 6.9e-05]	2.0e-05 [1.3e-05; 3.0e-05]	<0.001***

8742	1.1e-04 [8.7e-05; 1.5e-04]	3.9e-05 [1.3e-05; 6.6e-05]	4.7e-05 [2.9e-05; 8.4e-05]	7.2e-05 [4.4e-05; 1.0e-04]	2.6e-05 [9.3e-06; 5.0e-05]	5.9e-05 [1.7e-05; 9.0e-05]	5.7e-05 [2.1e-05; 1.5e-04]	6.2e-05 [4.4e-05; 8.1e-05]	4.9e-05 [3.3e-05; 6.9e-05]	5.7e-05 [1.5e-05; 1.5e-04]	<0.001***
9956	1.4e-04 [1.2e-04; 1.7e-04]	7.4e-05 [4.0e-05; 1.1e-04]	9.9e-05 [7.8e-05; 1.3e-04]	9.3e-05 [7.4e-05; 1.1e-04]	5.2e-05 [3.1e-05; 7.9e-05]	6.6e-05 [3.1e-05; 1.1e-04]	4.4e-05 [2.3e-05; 7.0e-05]	6.0e-05 [4.3e-05; 8.2e-05]	6.7e-05 [5.2e-05; 8.8e-05]	5.0e-05 [2.5e-05; 7.6e-05]	<0.001***
10096	2.3e-04 [5.2e-05; 4.0e-04]	9.1e-05 [4.2e-05; 1.7e-04]	8.2e-05 [4.6e-05; 9.8e-05]	2.6e-04 [1.7e-04; 4.8e-04]	6.1e-05 [3.3e-05; 1.2e-04]	9.1e-05 [5.4e-05; 1.1e-04]	5.8e-05 [3.1e-05; 1.0e-04]	5.5e-05 [4.2e-05; 8.8e-05]	5.5e-05 [3.7e-05; 7.4e-05]	3.5e-05 [1.7e-05; 4.2e-05]	<0.001***
10116	1.5e-04 [6.1e-05; 1.9e-04]	6.7e-05 [3.6e-05; 1.3e-04]	6.8e-05 [2.8e-05; 8.9e-05]	1.5e-04 [1.2e-04; 2.2e-04]	3.6e-05 [1.6e-05; 7.2e-05]	4.4e-05 [2.0e-05; 7.5e-05]	3.4e-05 [1.4e-05; 7.2e-05]	6.5e-05 [4.6e-05; 7.8e-05]	5.7e-05 [3.0e-05; 8.2e-05]	1.0e-05 [9.4e-06; 2.0e-05]	<0.001***
10437	7.1e-05 [3.1e-05; 1.3e-04]	6.8e-05 [3.6e-05; 1.3e-04]	9.4e-05 [5.8e-05; 1.8e-04]	1.7e-04 [1.3e-04; 2.6e-04]	9.5e-05 [4.8e-05; 1.8e-04]	1.3e-04 [7.8e-05; 2.7e-04]	1.1e-04 [5.5e-05; 2.1e-04]	2.0e-04 [1.4e-04; 3.0e-04]	1.6e-04 [1.0e-04; 2.6e-04]	1.3e-04 [1.0e-04; 2.2e-04]	<0.001***
10760	8.3e-05 [3.0e-05; 2.0e-04]	9.2e-05 [1.6e-05; 1.7e-04]	1.4e-04 [7.4e-05; 2.0e-04]	1.5e-04 [2.9e-05; 2.2e-04]	8.0e-05 [1.8e-05; 3.6e-04]	9.5e-05 [5.2e-05; 1.8e-04]	7.1e-05 [2.9e-05; 1.3e-04]	1.6e-04 [1.2e-04; 2.1e-04]	5.5e-05 [2.5e-05; 1.0e-04]	1.5e-04 [1.2e-04; 2.8e-04]	<0.001***
10837	2.2e-04 [2.0e-04; 4.7e-04]	1.8e-04 [1.1e-04; 5.2e-04]	1.7e-04 [7.8e-05; 2.7e-04]	1.8e-04 [1.2e-04; 4.9e-04]	3.0e-04 [1.7e-04; 6.8e-04]	7.7e-05 [6.1e-05; 9.7e-05]	8.5e-05 [3.8e-05; 1.3e-04]	5.0e-05 [3.1e-05; 6.8e-05]	5.8e-05 [3.5e-05; 7.8e-05]	3.2e-05 [1.5e-05; 5.9e-05]	<0.001***
11011	5.5e-05 [5.7e-06; 7.7e-05]	8.6e-05 [2.7e-05; 1.3e-04]	2.4e-04 [1.6e-04; 3.1e-04]	3.3e-05 [1.3e-05; 7.7e-05]	1.3e-05 [4.3e-08; 6.6e-05]	1.5e-04 [1.1e-04; 2.4e-04]	2.7e-05 [9.2e-06; 6.5e-05]	4.7e-05 [3.1e-05; 6.6e-05]	7.2e-05 [4.7e-05; 1.0e-04]	4.8e-05 [1.7e-05; 6.5e-05]	<0.001***
11725	2.9e-04 [1.2e-04; 4.1e-04]	1.2e-04 [5.2e-05; 2.5e-04]	3.5e-04 [1.8e-04; 4.1e-04]	2.3e-04 [1.2e-04; 2.8e-04]	1.2e-04 [5.9e-05; 2.0e-04]	2.9e-04 [2.0e-04; 3.6e-04]	1.3e-04 [7.6e-05; 2.3e-04]	3.1e-04 [2.2e-04; 4.7e-04]	1.6e-04 [8.8e-05; 2.4e-04]	9.3e-05 [6.7e-05; 2.6e-04]	<0.001***
14692	6.0e-04 [3.1e-04; 6.9e-04]	3.3e-04 [1.6e-04; 6.6e-04]	3.8e-04 [1.9e-04; 5.5e-04]	3.0e-04 [2.5e-04; 4.0e-04]	2.6e-04 [1.8e-04; 5.3e-04]	2.6e-04 [1.2e-04; 3.4e-04]	1.8e-04 [9.9e-05; 4.1e-04]	7.7e-05 [5.5e-05; 1.0e-04]	8.1e-05 [5.4e-05; 1.3e-04]	1.4e-04 [1.3e-04; 2.6e-04]	<0.001***

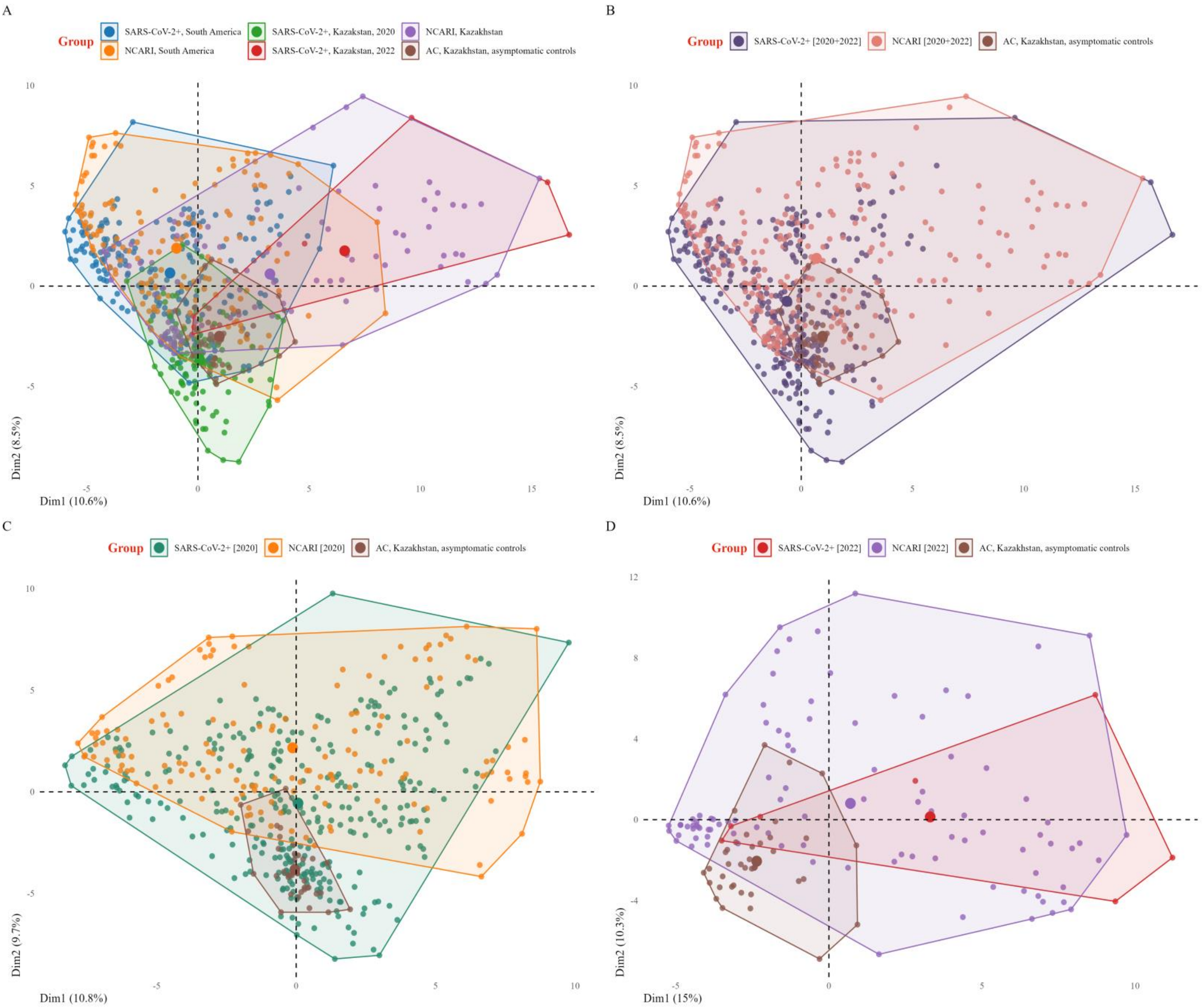

Figure S7. PCA of the mass spectra of all sub-groups from the combined dataset based on the peak intensity matrix for Analysis II.

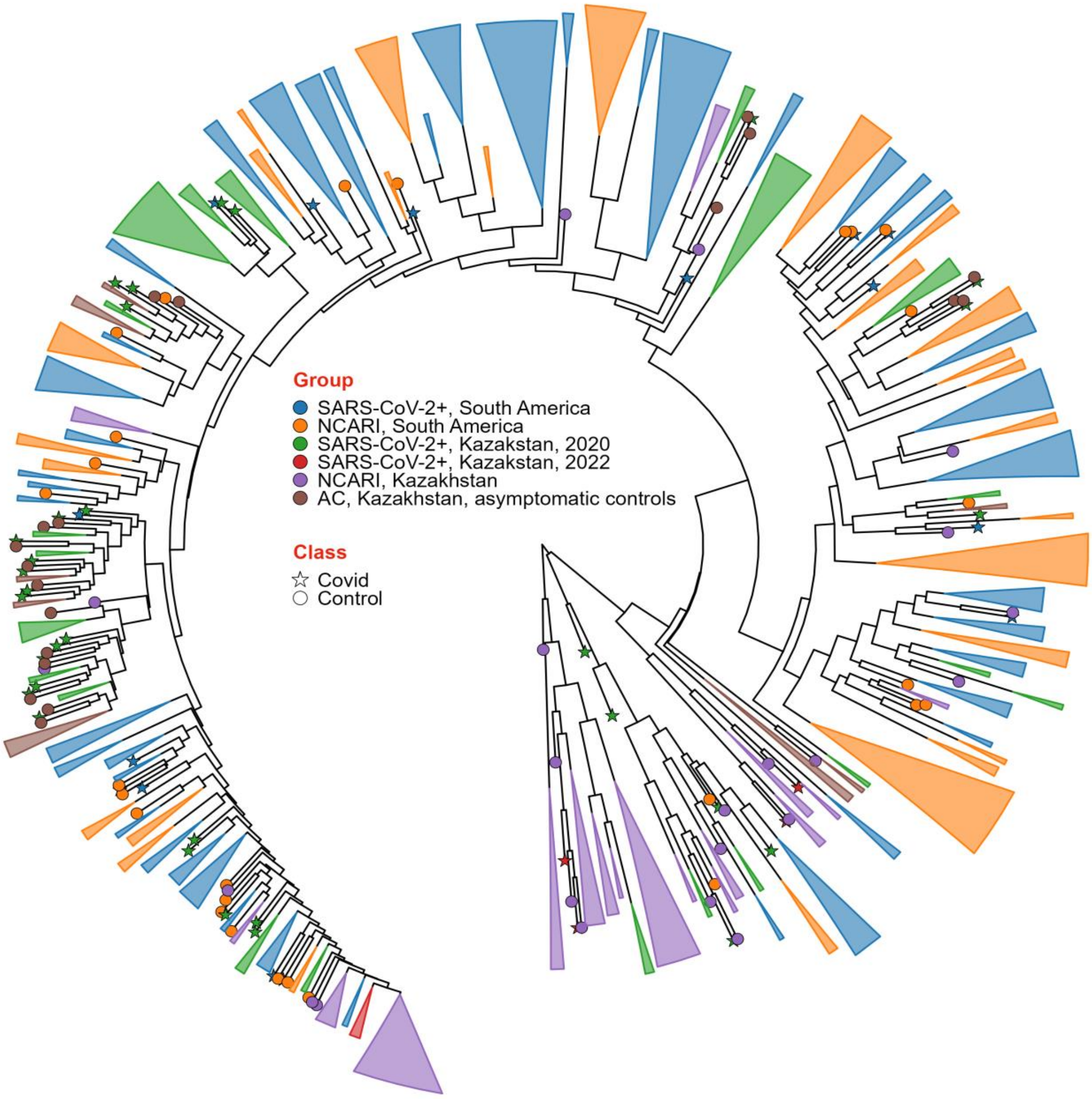

Figure S8. Dendrogram of the mass spectra stratified by sub-group from the combined dataset based on the peak intensity matrix for Analysis II.

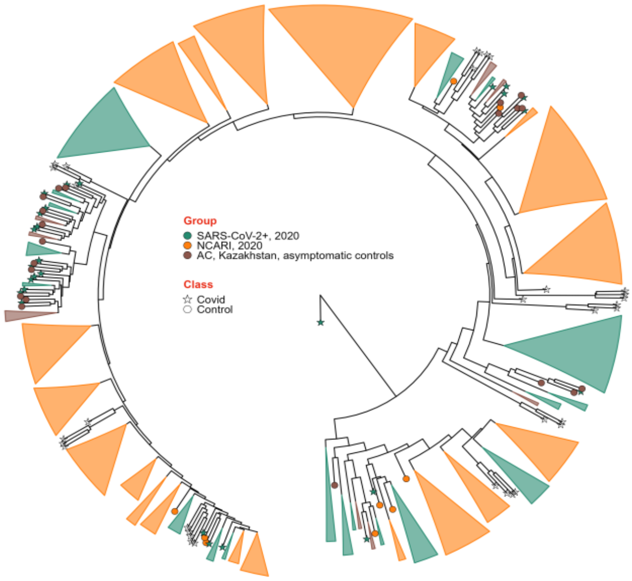

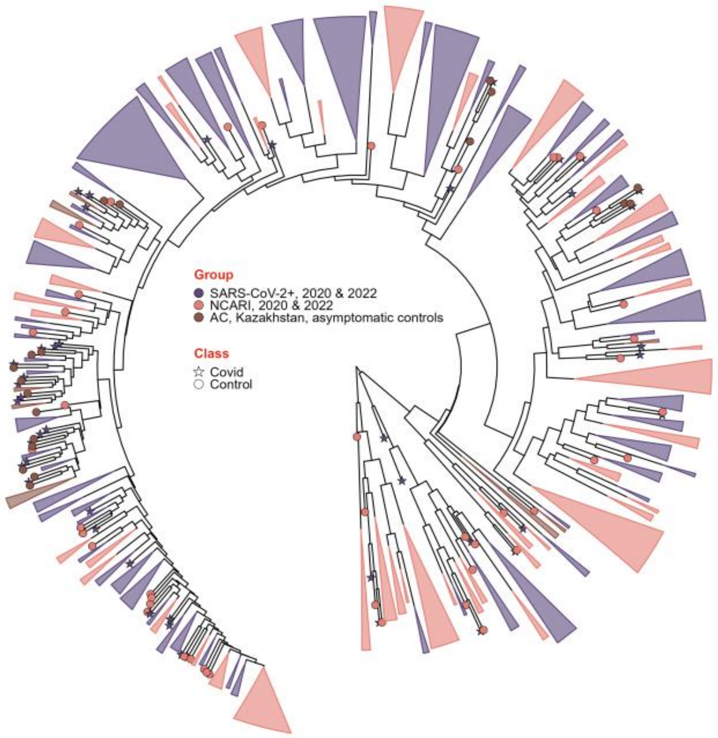

Figure S9. Dendrogram of the mass spectra stratified by sub-group from the combined dataset based on the peak intensity matrix for Analysis II.

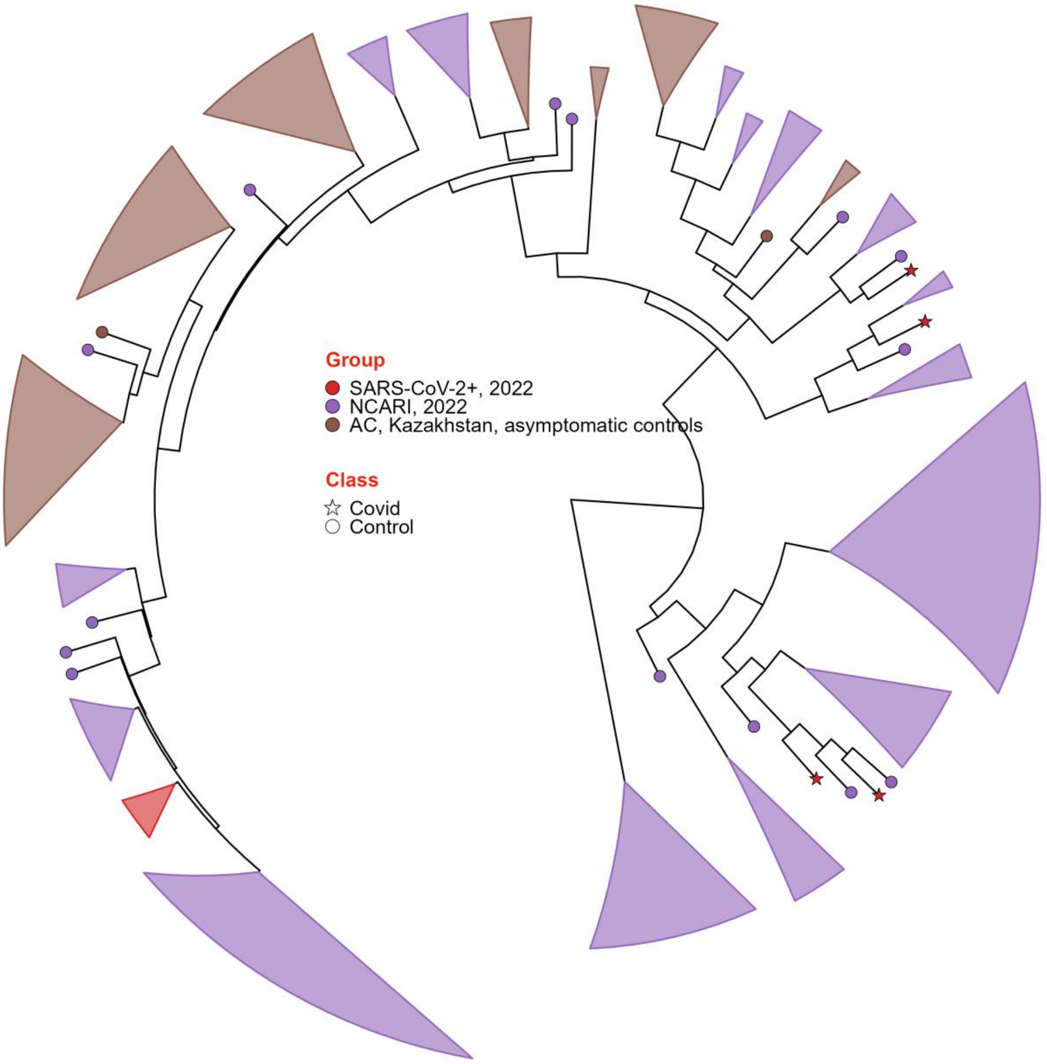

98

99 **Figure S10.** Dendrogram of the mass spectra stratified by sub-group from the combined dataset based on the
100 peak intensity matrix for Analysis II.

Table S6. ROC AUC values of 7 ML models trained in Analysis II to differentiate each sub-group within the Kazakhstan (KZ) dataset. Median [25%; 75%]. Each model was tested 5 times on a 20% subset of Kazakhstan samples (the rest 80% were used to train the ML models). NCARI= non-COVID acute respiratory infection. AC=asymptomatic controls.

Paired comparisons	DT	KNN	NB	RF	SVM-L	SVM-R	XGBoost
SARS-CoV-2 vs rest	0.972	0.92	0.801	0.93	0.94	0.983	0.958
	[0.966; 0.979]	[0.88; 0.92]	[0.769; 0.808]	[0.90; 0.96]	[0.93; 0.95]	[0.958; 0.987]	[0.943; 0.970]
NCARI vs rest	0.996	0.95	0.90	0.984	0.964	0.995	0.986
	[0.979; 1.000]	[0.93; 0.97]	[0.83; 0.92]	[0.983; 1.000]	[0.954; 0.984]	[0.964; 0.996]	[0.981; 0.996]
AC vs rest	0.979	0.898	0.82	0.972	0.986	0.995	0.981
	[0.979; 0.984]	[0.886; 0.955]	[0.79; 0.85]	[0.966; 0.984]	[0.976; 0.992]	[0.990; 0.996]	[0.979; 0.988]
Micro-averaged	0.977	0.93	0.785	0.951	0.965	0.991	0.960
	[0.966; 0.981]	[0.90; 0.94]	[0.775; 0.834]	[0.925; 0.968]	[0.958; 0.966]	[0.965; 0.991]	[0.956; 0.977]
Macro-averaged	0.971	0.91	0.806	0.962	0.961	0.983	0.972
	[0.965; 0.981]	[0.90; 0.92]	[0.794; 0.857]	[0.941; 0.972]	[0.955; 0.961]	[0.955; 0.986]	[0.951; 0.977]
